# Loss of Dendritic Complexity Precedes Neurodegeneration in a Mouse Model with Disrupted Mitochondrial Distribution in Mature Dendrites

**DOI:** 10.1016/j.celrep.2016.09.004

**Published:** 2016-10-04

**Authors:** Guillermo López-Doménech, Nathalie F. Higgs, Victoria Vaccaro, Hana Roš, I. Lorena Arancibia-Cárcamo, Andrew F. MacAskill, Josef T. Kittler

**Affiliations:** 1 Department of Neuroscience, Physiology and Pharmacology, University College London, Gower Street, London WC1E 6BT, UK

**Keywords:** dendritic development, mitochondrial transport, neuronal polarity

## Abstract

Correct mitochondrial distribution is critical for satisfying local energy demands and calcium buffering requirements and supporting key cellular processes. The mitochondrially targeted proteins Miro1 and Miro2 are important components of the mitochondrial transport machinery, but their specific roles in neuronal development, maintenance, and survival remain poorly understood. Using mouse knockout strategies, we demonstrate that Miro1, as opposed to Miro2, is the primary regulator of mitochondrial transport in both axons and dendrites. Miro1 deletion leads to depletion of mitochondria from distal dendrites but not axons, accompanied by a marked reduction in dendritic complexity. Disrupting postnatal mitochondrial distribution in vivo by deleting Miro1 in mature neurons causes a progressive loss of distal dendrites and compromises neuronal survival. Thus, the local availability of mitochondrial mass is critical for generating and sustaining dendritic arbors, and disruption of mitochondrial distribution in mature neurons is associated with neurodegeneration.

## Highlights

• Miro1 deletion alters mitochondrial distribution in dendrites but not axons
• Mitochondrial distribution impacts dendritic development
• Correct mitochondrial distribution is key to maintain complex dendritic geometries
• Aberrant dendritic mitochondrial distribution triggers neurodegeneration

## Graphical Abstract

**Figure:**
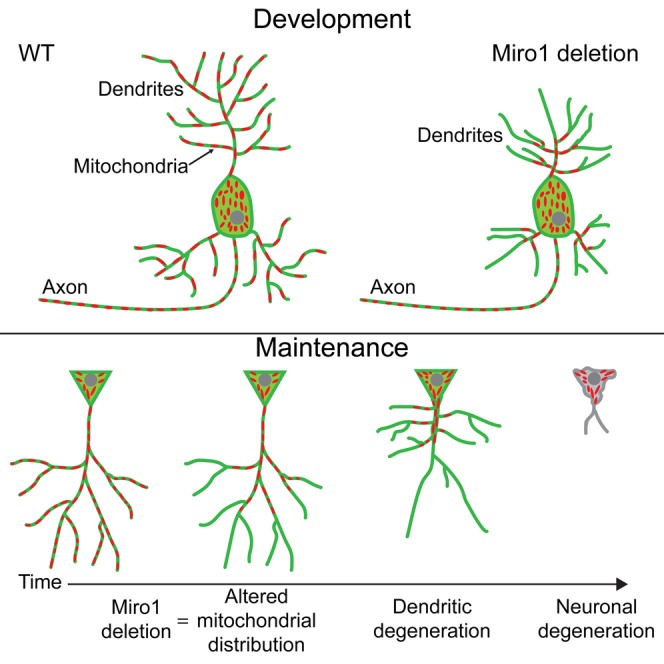

## Teaser

Complex dendritic morphologies are essential for neural circuit formation and brain computation. López-Doménech et al. demonstrate that Miro1-dependent dendritic mitochondrial positioning critically regulates the development of the dendritic tree and sustains arborization. Disrupting mitochondrial distribution in mature neurons leads to the loss of dendritic complexity, which precedes neurodegeneration.

## Introduction

Although only 2% of the body’s weight, the brain consumes 20% of the body’s resting energy production (Harris et al., 2012), which may rise to 50% in the developing brain (Kuzawa et al., 2014). Energy in the adult brain is mainly used to reverse the ion influxes underlying synaptic and action potential signaling, for pumping neurotransmitters and for supporting signal transduction and trafficking events during synaptic plasticity (Harris et al., 2012, Rangaraju et al., 2014). During brain development energy may also be required to fuel the extensive neuronal growth necessary to build the complex neuronal morphologies (Kuzawa et al., 2014) that are essential for the formation of neuronal circuits (Jan and Jan, 2010) and for brain computation (Eyal et al., 2014, Spruston, 2008). While changes in dendritic complexity have been reported to occur in a number of neurological and neurodegenerative diseases or during normal aging (Grutzendler et al., 2007, Kabaso et al., 2009, Kulkarni and Firestein, 2012), the energy costs and cellular mechanisms underlying dendritic sustainability are poorly understood.

Mitochondria are critical for ATP provision and play other essential roles in neurons such as buffering calcium (MacAskill and Kittler, 2010, Sheng and Cai, 2012). The very large size of many neurons suggests that mitochondrial distribution must be spatially matched to local energy usage and calcium buffering requirements during the growth and maintenance of axons and dendrites. Indeed, alterations in mitochondrial dynamics compromise synaptic function (MacAskill and Kittler, 2010), and defective mitochondrial trafficking has been related to neurological and neurodegenerative disease (MacAskill et al., 2010, Sheng and Cai, 2012). Therefore, mitochondrial distribution and trafficking may be key determinants for both the generation and the long-term maintenance of the complex neuronal morphologies essential for brain information processing (MacAskill and Kittler, 2010, Sheng and Cai, 2012). Currently, however, the relationship between mitochondrial distribution and the morphogenesis and maintenance of neuronal architecture in vivo remains unclear.

Miro (mitochondrial Rho) GTPases are central regulators of mitochondrial trafficking that act as adaptors, linking mitochondria to motor proteins (Birsa et al., 2013, Fransson et al., 2006). Here, we use mouse genetics to investigate the differential role of Miro1 and Miro2 for mitochondrial trafficking, neuronal morphogenesis, and the maintenance of complex neuronal architecture. We show that Miro1 is the main regulator of mitochondrial trafficking and distribution. Depleting Miro1 levels in vivo during development disrupts neuronal morphogenesis, while postnatal Miro1 disruption in mature neurons leads to a loss of dendritic complexity starting in the distal dendrites. Our findings demonstrate that altering mitochondrial positioning in dendrites leads to a progressive loss of dendritic complexity, which, in turn, triggers neuronal death in a way that closely resembles a neurodegenerative process.

## Results

### Miro1 and Miro2 Have Different Roles for Animal Viability and Mitochondrial Trafficking

To study the roles of Miro1 and Miro2, we characterized constitutive mouse knockout strains for *Rhot1* (Miro1 gene) and *Rhot2* (Miro2 gene) (Figures S1A–S1D) (Skarnes et al., 2011). Protein levels within brain lysates confirmed the specific deletion of Miro1 and Miro2 proteins, respectively (Figure S1E). *Rhot2* knockout animals (Miro2^KO^ hereafter) were viable and fertile, whereas *Rhot1* knockout animals (Miro1^KO^) were born alive at the expected Mendelian ratios but remained cyanotic and died within the first 15 to 30 min of life (Nguyen et al., 2014).

**Figure 1:**
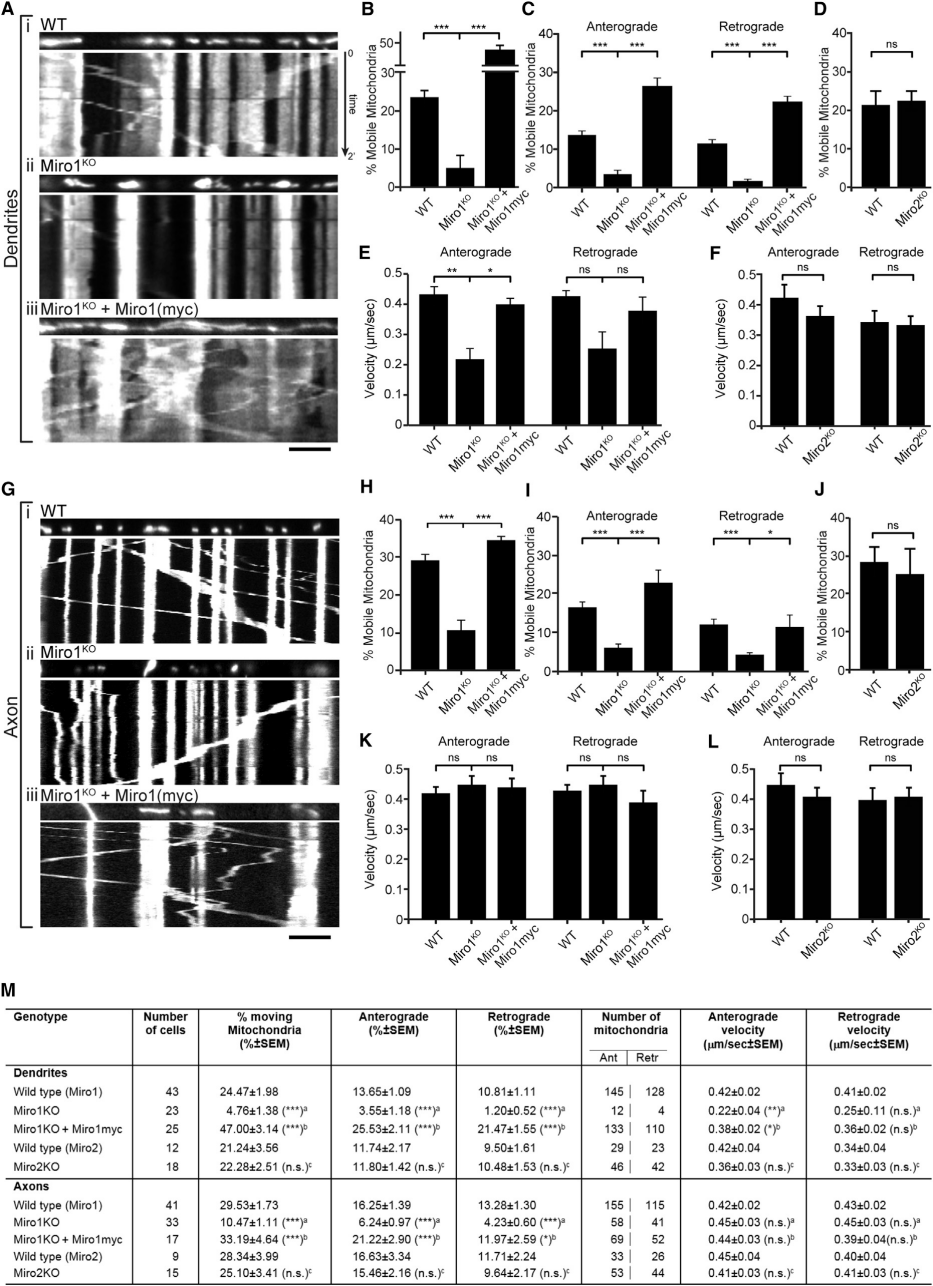
Miro1, Not Miro2, Is the Main Regulator of Mitochondrial Transport (A and G) Images and kymographs from dendrites (A) and axons (G) of hippocampal neurons from (1) WT, (2) Miro1^KO^, and (3) with Miro1-myc rescue in Miro1^KO^ cultures. Images show mitochondria at time = 0 and corresponding kymographs show their motility over a 2-min period (height). Scale bar, 10 μm. (B and H) Percentage of mobile mitochondria in dendrites (B) and axons (H) (dendrites WT = 43, Miro1^KO^ = 23, Miro1^KO^ + Miro1myc = 17, axons WT = 41, Miro1^KO^ = 33, Miro1^KO^ + Miro1myc = 15). (C and I) Percentage of mitochondria moving in the anterograde or retrograde direction in dendrites (C) and axons (I) of WT, Miro1^KO^, and Miro1^KO^ + Miro1myc neurons. (D and J) Percentage of mobile mitochondria in WT and Miro2^KO^ dendrites (D) and axons (J) (dendrites WT = 12, Miro2^KO^ = 18, axons WT = 9, Miro2^KO^ = 15). (E, F, K, and L) Average velocity of moving mitochondria from Miro1 (E and K) or Miro2 (F and L) experiments (dendritic mitochondria in (E): WT = 273, Miro1^KO^ = 16, Miro1^KO^ + Miro1myc = 243, and in (F) WT = 52, Miro2^KO^ = 88); (axonal mitochondria in (K) WT = 270, Miro1^KO^ = 101, Miro1^KO^ + Miro1myc = 121, and in (L) WT = 59, Miro2^KO^ = 97). (M) Mitochondrial trafficking measured in 14-DIV hippocampal cultured neurons: (^a^) compared to wild-type (Miro1 experiments); (^b^) compared to Miro1^KO^ (Miro1 experiments); (^c^) compared to wild-type (Miro2 experiments). Statistical differences were calculated assuming non-parametric distributions. ^∗^p < 0.05, ^∗∗^p < 0.01, and ^∗∗∗^p < 0.001. Error bars are SEM.

To address the specific roles of Miro1 and Miro2 for mitochondrial trafficking, we compared hippocampal neuronal cultures from individual wild-type (WT) or knockout E16 embryos generated by heterozygous (Miro1^+/−^ X Miro1^+/−^ or Miro2^+/−^ X Miro2^+/−^) matings. In both 6–7 days in vitro (DIV) and 14–15 DIV Miro1^KO^ neurons expressing GFP to fill the cell and MtdsRed2 to label mitochondria, anterograde and retrograde mitochondrial trafficking was altered in dendrites (∼85% decrease) and in axons (∼65% decrease) (Figures 1 and S3A–S3D). The velocity of the remaining motile mitochondria at 14–15 DIV was unaltered in axons but was reduced by ∼50% in the anterograde direction within dendrites whereas retrograde velocity was unaffected (Figures 1E, 1K, and 1M). Interestingly, the small amount of mitochondrial trafficking that remained was still sensitive to neuronal activation induced by glutamate (Macaskill et al., 2009) indicating that other mechanisms exist that can sense Ca^2+^ and induce Miro1-independent mitochondrial stopping (Figures S1F–S1I). Mitochondrial trafficking was fully rescued by expression of Miro1-myc (Figure 1) but only partially by Miro2 expression (Figures S2A and S2B). Unexpectedly, Miro2 deletion (Miro2^KO^ neurons) had no substantial effect on mitochondrial trafficking (Figures 1 and S1J) suggesting Miro2 is not the main regulator of mitochondrial trafficking or that its function can be compensated by Miro1. Moreover, the trafficking of Rab5GFP positive early endosomes (Figure S1K) and axonal retrograde transport of Rab7GFP positive signaling endosomes (Deinhardt et al., 2006) was unaffected in Miro1^KO^ neurons (Figures S1L–S1N) further confirming the critical importance and specificity of Miro1 deletion for mitochondrial trafficking.

### Disruption of Dendritic Mitochondrial Distribution upon Miro1 Deletion

**Figure 2:**
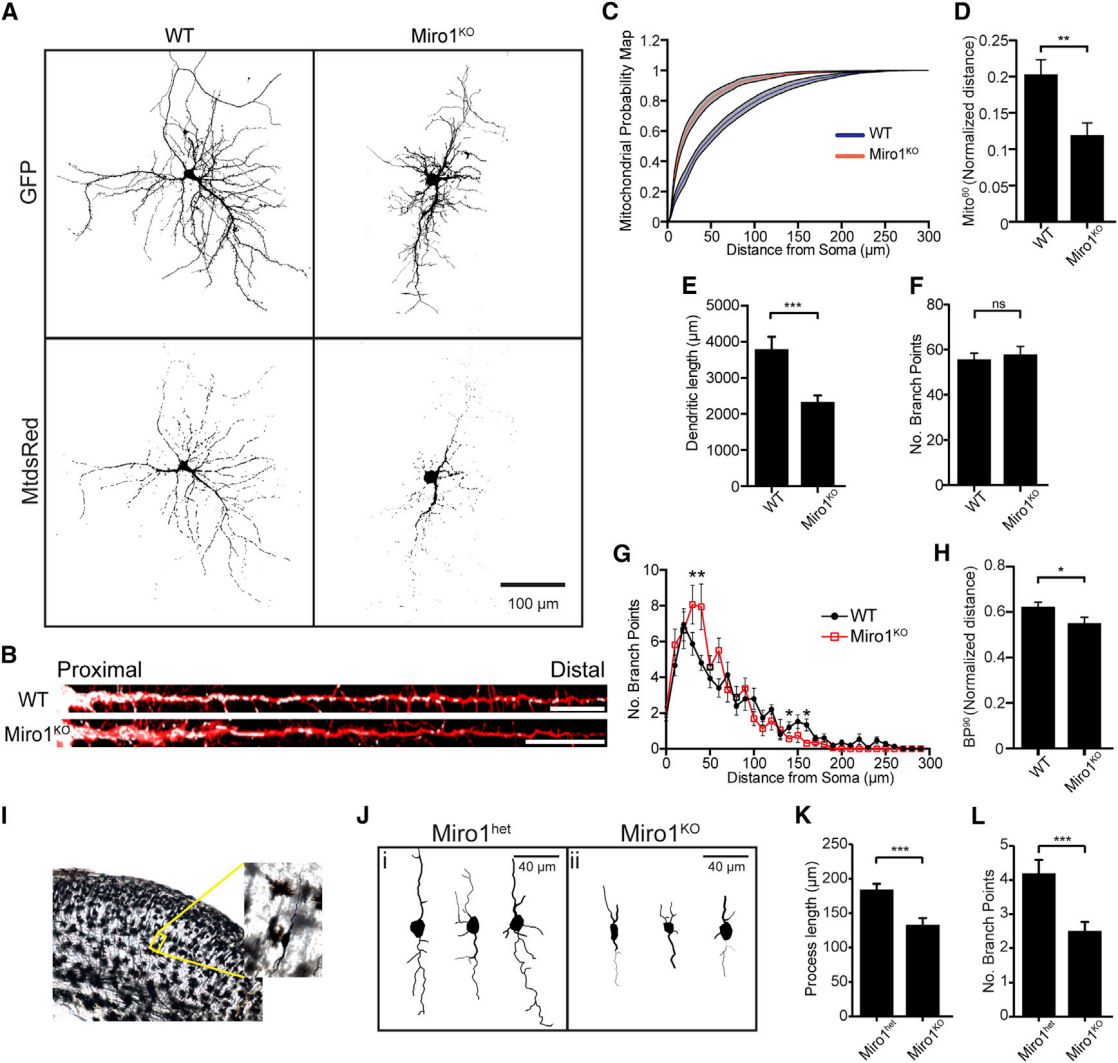
Miro1-Dependent Mitochondrial Distribution Drives Dendritic Morphogenesis and Arborization (A) WT and Miro1^KO^ neurons expressing GFP and MtdsRed2 and imaged at 14 DIV (scale bar, 100 μm). (B) Scaled dendritic process (red) showing mitochondrial distribution (white) in WT and Miro1^KO^ dendrites (scale bar, 30 μm). (C and D) Mitochondrial probability map (MPM) (C) and the length-normalized Mito^60^ value from WT and Miro1^KO^ neurons (D) (number of cells: WT = 11 and Miro1^KO^ = 17). (E–H) (E) Dendritic length and (F) number of branchpoints from WT and Miro1^KO^ neurons. Sholl analysis of branchpoint number (G) and the length-normalized BP^90^ value (H) (number of cells: WT = 15 and Miro1^KO^ = 19). (I) Golgi staining of E18 brains and example traces (J) of E18 cortical neurons from Miro1^KO^ and Miro1^het^ control littermates. Process length (K) and number of branchpoints (L) from the traced neurons (n = 35 cells for each genotype). ^∗^p < 0.05, ^∗∗^p < 0.01, ^∗∗∗^p < 0.001. Error bars are SEM.

We found that in Miro1^KO^ neurons the vast majority of mitochondria were accumulated in proximal regions of dendrites and only sparsely distributed within distal dendrites, with large dendritic segments almost entirely devoid of mitochondria (Figures 2A and 2B). To quantify this effect, we performed a mitochondrial “Sholl” distribution analysis (see Experimental Procedures), to give a “mitochondrial probability map” (MPM): the cumulative probability plot of mitochondrial distribution as a function of distance from the soma. The MPM shows a clear shift to the left in the curve for Miro1^KO^ neurons at both 6 DIV and 14–15 DIV, confirming an accumulation of mitochondria in the proximal dendrites of Miro1^KO^ cells and reduction in distal dendrites (Figures 2C and S3E). To account for variability in neuronal process length, we defined a normalized value, Mito^60^, the point at which 60% of the total mitochondrial mass is found within a normalized proximal to distal dendritic axis (where the dendritic tip is defined as 1). In Miro1^KO^ neurons the Mito^60^ value was decreased by ∼40% at both 14–15 DIV (p = 0.003) and 6–7 DIV (p = 0.035), confirming a more accumulated distribution of mitochondria within proximal dendrites (Figures 2D and S3F). In contrast, in Miro2^KO^ neurons the MPM and Mito^60^ values were unaltered (Figures S2C and S2D, p = 0.16). Importantly, mitochondria accumulated in proximal Miro1^KO^ dendrites were metabolically active and kept their mitochondrial membrane potential, as revealed by tetramethylrhodamine methyl ester (TMRM) staining (Figures S3K and S3L). Thus, Miro1 control of mitochondrial trafficking is a key determinant of correct mitochondrial distribution within dendrites—a function that cannot be readily compensated by endogenous Miro2.

### Miro1-Dependent Mitochondrial Trafficking and Distribution Are Critical for Correct Dendritic Development In Vitro and In Vivo

Neurons lacking Miro1 appeared smaller and less developed than control neurons at 14–15 DIV (Figure 2A). Total dendritic length was ∼35% decreased in Miro1^KO^ neurons (Figure 2E, WT 3793 ± 89 μm, Miro1^KO^ 2447 ± 54 μm, p = 0.0003), although surprisingly the total number of branchpoints was unaffected (Figure 2F, WT 55.7 ± 2.78, Miro1^KO^ 57.9 ± 3.53, p = 0.63). Dendritic Sholl analysis of Miro1^KO^ neurons also revealed a marked increase in dendritic complexity in proximal regions and a decrease distally, closely correlating with the observed changes in mitochondrial distribution (Figure 2G). To account for changes in neuronal size, we defined a value that represented where 90% of the branchpoints are found within size-normalized cells (BP^90^). The BP^90^ for Miro1^KO^ neurons is significantly less than for WT neurons as Miro1^KO^ neurons have significantly more branchpoints near the soma (Figure 2H). In contrast Miro2^KO^ neurons appeared similar to WT cells in terms of dendritic architecture (Figures S2E–S2H; dendritic length, p = 0.85; total branchpoints, p = 0.62) indicating that in the presence of Miro1, Miro2 is not required for correct dendritic development. Younger Miro1^KO^ neurons (6–7 DIV) were similar in terms of dendritic length and number of primary dendrites and branchpoints compared to WT (Figures S3G–S3I). Interestingly, Sholl analysis demonstrated that they were already beginning to show less distal complexity (120 μm outward from the soma) compared to WT neurons (Figure S3J). Moreover, reconstructions of Golgi-stained neurons (Figure 2I) located in the lower levels of the cortical plate of Miro1^KO^ brains showed a clear reduction in neurite length (p = 0.0009) and branchpoint number (p = 0.0005) (Figures 2J–2L) at an early stage of cortex development (at E18, when Miro1^KO^ animals are viable) supporting a role for Miro1 in regulating neuronal morphogenesis in vivo. Thus local availability of mitochondrial mass is essential for correct neuronal process development.

### Miro1 Is Necessary for Maintaining Mitochondrial Distribution and Dendritic Complexity in Mature Neurons In Vivo

To test whether the loss of Miro1 altered mitochondrial distribution in vivo and impacts postnatal dendritic maintenance, we generated a conditional Miro1 allele (Miro1^lox/lox^, see Figure S1A) and crossed this with a strain expressing Cre recombinase under the Ca^2+^/calmodulin-dependent protein kinase-alpha specific promoter (CaMKIIα-Cre), which has been widely used to drive Cre-mediated genetic deletion in postnatal forebrain. Miro1^Δ/Δ,Cre(+/−)^ animals (hereafter Miro1^CKO^) developed normally and appeared indistinguishable from their Miro1^lox/lox,Cre(−/−)^ (WT hereafter) control littermates. Specific deletion of the Miro1 gene in Miro1^CKO^ animals occurred primarily in adult principal neurons of the cortex and hippocampus (Figure S4A). Interestingly, whereas, in WT animals Miro2 levels were progressively decreased over time (Figure S4B), this was not observed in brains deleted for Miro1 (Figures S4D and S4E) suggesting that Miro2 may be upregulated to counteract the depletion of Miro1.

**Figure 3:**
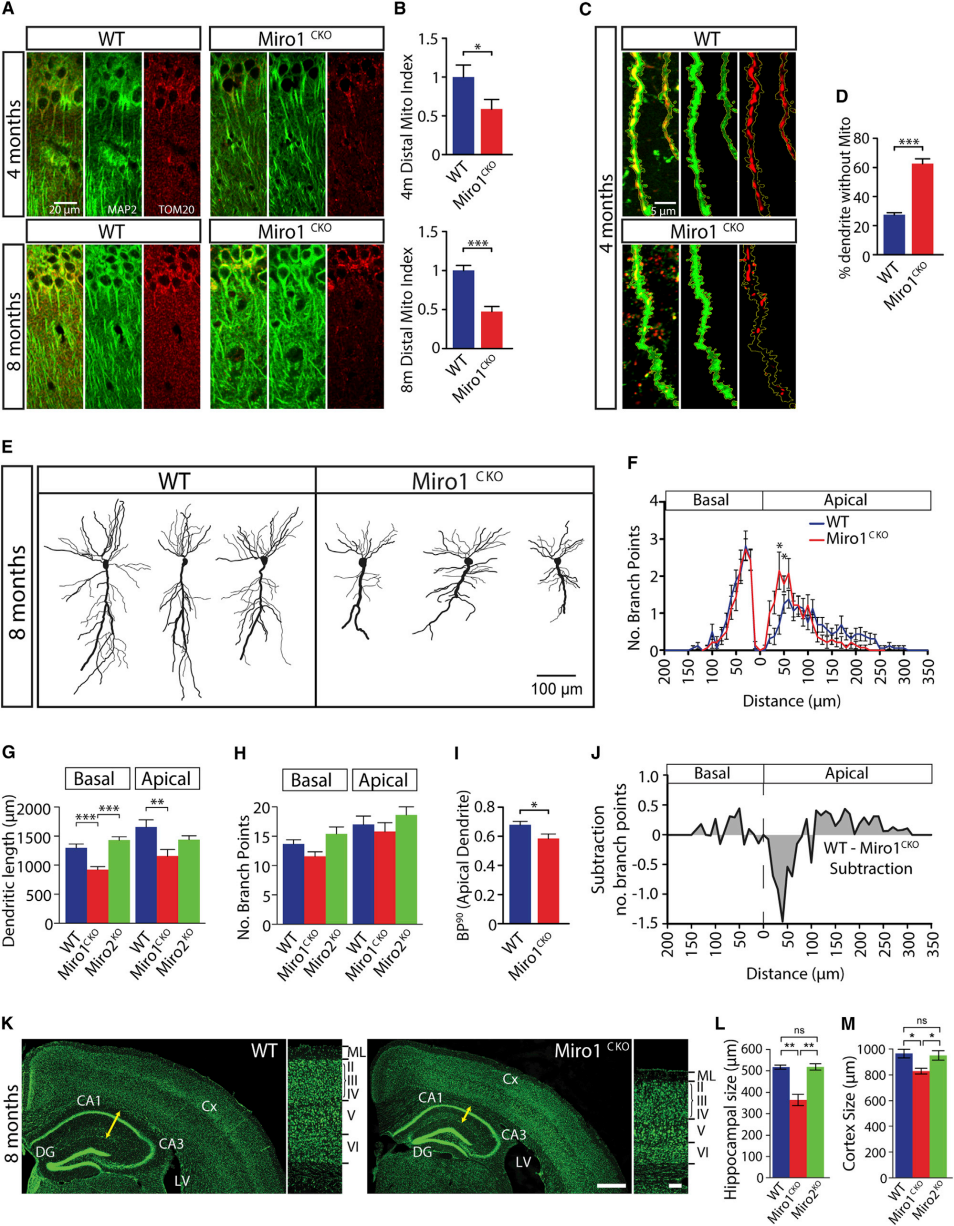
Requirement of Miro1 for Dendritic Maintenance (A and B) Mitochondrial distribution in WT and Miro1^CKO^ CA1 neurons at 4 and 8 months of age (A) and normalized mitochondrial index (B) (n = images: at 4 months WT = 23, Miro1^CKO^ = 23; at 8 months WT = 22, Miro1^CKO^ = 20; t test). (C and D) Dendritic segments from the stratum lacunosum-moleculare of AAV infected animals at 4 months (C). Quantification of the dendritic length not occupied by mitochondria (D) in WT and Miro1^CKO^. (E–J) Reconstructed traces (E), branchpoint Sholl analysis (F), dendritic length (G), and number of branchpoints (H) from WT and Miro1^CKO^ Golgi-stained CA1 neurons at 8 months of age. (I) Normalized BP^90^ value for branching along the apical dendrite. (J) Subtracted (WT-Miro1^CKO^) plot of the Sholl distribution (n = cells: WT = 15, Miro1^CKO^ = 14; ANOVA-NK). (K) FluoroNissl staining of brain coronal sections showing the hippocampus, cortex, and lateral ventricles of 8-month-old WT and Miro1^CKO^ brains (scale bar, 500 μm). Right panels show the structure of the cerebral cortex in the somatosensory area (scale bar, 100 μm). (L and M) Quantification of hippocampal (yellow arrows) (L) and cortical thickness (M) from brains at 8 months (n = animals: WT = 4, Miro1^CKO^ = 4, Miro2^KO^ = 4; ANOVA-NK). ^∗^p < 0.05, ^∗∗^p < 0.01, ^∗∗∗^p < 0.001. Error bars are SEM.

By immunostaining with the mitochondrial marker Tom20 we observed in 4-month-old Miro1^CKO^ brains a decrease in the mitochondrial index (mitochondrial area/MAP2 positive dendritic area) in distal regions of the CA1 neurons corresponding to the Stratum Lacunosum-Moleculare (SLM), compared to WT (Figures 3A and 3B, p = 0.043; t test). At 8 months, Tom20 staining revealed an even more enhanced loss of mitochondrial density in Miro1^CKO^ CA1 distal dendrites (Figures 3A and 3B; p < 0.001; t test). To ensure that the loss in mitochondrial density was specifically in the dendrites and not in the axonal projections that populate the SLM region, we performed intracerebral injections of non-replicating Adeno-Associated Virus (AAV2) encoding MtDsRed2 and GFP (Stephen et al., 2015) into the CA1-CA3 region of the hippocampus. Neurons from control animals showed mitochondria distributed homogeneously throughout the dendritic length, whereas Miro1^CKO^ neurons presented long segments of distal dendrites devoid of mitochondria (Figures 3C and 3D, percentage of dendritic length without mitochondria; WT 25% ± 2%, Miro1^CKO^ 65% ± 5%; p < 0.001, t test), indicating that, also in vivo, Miro1 regulates mitochondrial distribution in mature dendrites.

Although dendritic mitochondrial distribution was already affected at 4 months of age, no defects in dendritic complexity were apparent when comparing Golgi-stained CA1 pyramidal neurons from WT and Miro1^CKO^ brains (Figures S5B–S5E: dendritic length: basal; WT 1321 ± 42 μm, Miro1^CKO^ 1394 ± 69 μm, apical; WT 1183 ± 31 μm, Miro1^CKO^ 1129 ± 86 μm; branchpoints: basal; WT 16.7 ± 0.5, Miro1^CKO^ 15.6 ± 0.8, apical; WT 16.3 ± 0.7, Miro1^CKO^ 14.8 ± 1.1; ANOVA and post hoc Newman-Keuls [ANOVA-NK]). In contrast, at 8 months, Miro1^CKO^ CA1 neurons showed a significant decrease in total dendritic length in both the basal and apical dendrites, (Figures 3E and 3G, basal: WT 1307 ± 100 μm, Miro1^CKO^ 889 ± 83 μm, p < 0.01; apical: WT 1686 ± 183 μm, Miro1^CKO^ 1155 ± 172 μm, p < 0.05; ANOVA-NK). Interestingly, while the number of branchpoints was similar to that of WT neurons (Figure 3H), Sholl analysis revealed more branchpoints in the proximal region of the apical dendrite and reduced branching in the distal regions in Miro1^CKO^ neurons (Figures 3F, 3I, and 3J). Age-matched Miro2^KO^ neurons showed no morphological defects (Figures 3G and 3H). The loss of dendritic complexity at 8 months in Miro1^CKO^ animals correlated with a clear decrease in brain size compared to WT (Figure S4C) and an increase in the size of the ventricles with a reduction in hippocampal size (Figures 3K and 3L, pial to apical surface distance of hippocampus: WT 516.8 ± 9.7 μm, Miro1^CKO^ 363.3 ± 26.7 μm, p < 0.01; and Miro2^KO^ 517.8 ± 14.9 μm, ANOVA-NK) and thickness of the cortex (Figures 3K and 3M, WT 960.4 ± 35.1 μm, Miro1^CKO^ 824.3 ± 24.4 μm, p < 0.05; and Miro2^KO^ 946.1 ± 35.4 μm, ANOVA-NK).

Therefore, the postnatal loss of Miro1 disrupts mitochondrial distribution in the proximal to distal dendritic axis leading to a gradual decrease in dendritic complexity particularly affecting the most distal dendrites.

### The Distribution of Mitochondria along the Axon Is Not Dependent on Miro1

**Figure 4:**
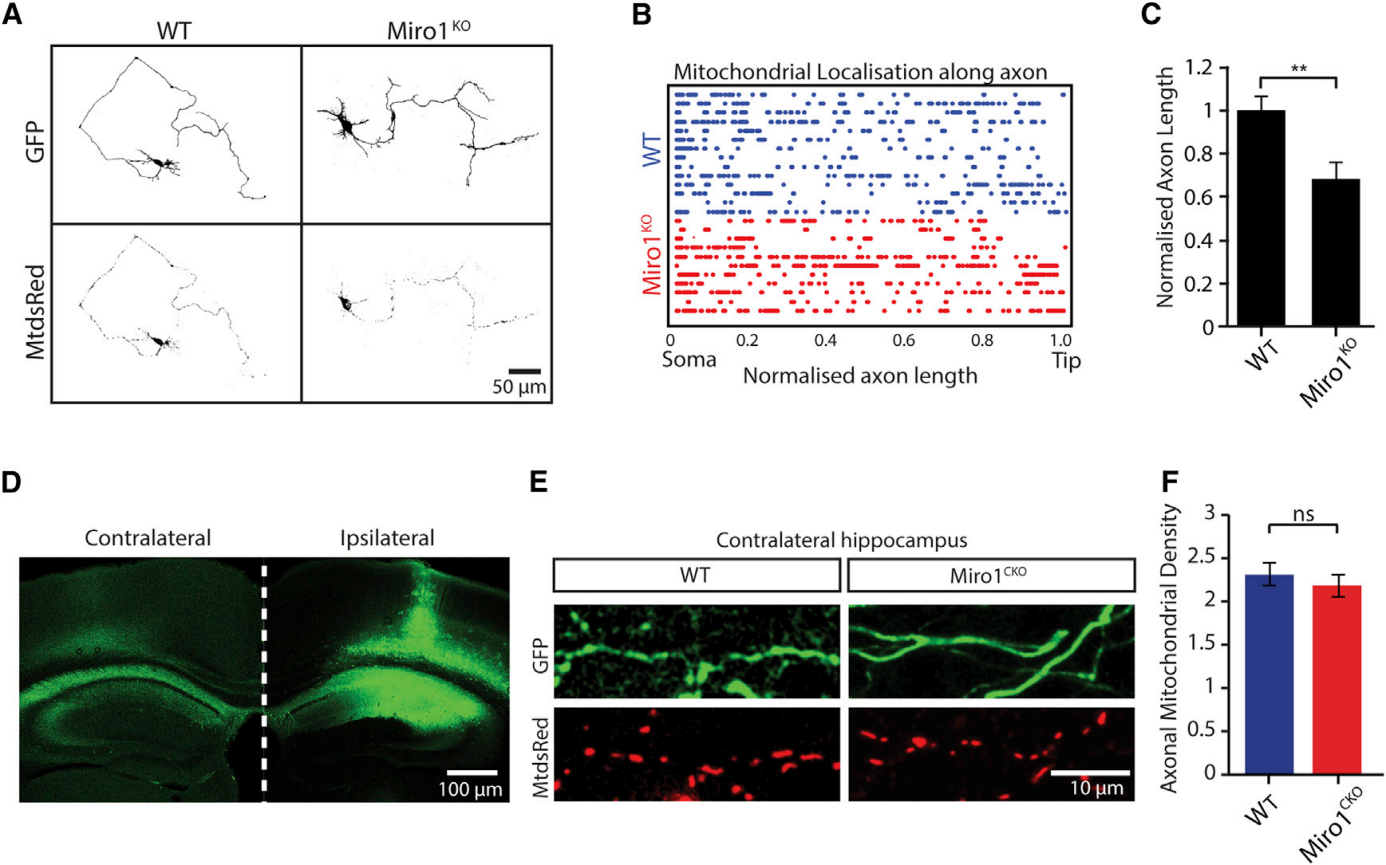
Mitochondrial Distribution along the Axon Is Independent of Miro1 (A) Stitched images (grayscale) showing the whole axonal length of WT and Miro1^KO^ neurons at 6 DIV (scale bar, 50 μm). (B) Plot of the relative positions of individual mitochondria over length-normalized axons. (C) Total axonal length from WT and Miro1^KO^ neurons (number of neurons, WT = 14, Miro1^KO^ = 11, t test). (D) Images of the contralateral and ipsilateral hemispheres of AAV2-injected brains. (E) Distribution of axonal mitochondria in the contralateral hippocampus from CA3 neurons infected with AAV2 encoding GFP and MtdsRed2. (F) Density of axonal mitochondria in WT and Miro1^CKO^ neurons (number of axons, WT = 17, Miro1^CKO^ = 15; number of animals, WT = 2, Miro1^CKO^ = 2). p < 0.05, ^∗∗^p < 0.01, ^∗∗∗^p < 0.001. Error bars are SEM.

As opposed to dendrites, mitochondria distributed homogeneously throughout axons of Miro1^KO^ cultured hippocampal neurons (Figure 4A). A plot of the relative mitochondrial positions along WT and Miro1^KO^ axons from 6-DIV neurons, transfected with GFP and MtdsRed2 and normalized for changes in length (Figure 4B), showed that mitochondria are distributed throughout the entire length of the axon, despite the fact that fast axonal mitochondrial trafficking is severely altered in Miro1^KO^ neurons. This suggests that other mechanisms can compensate the absence of Miro1 for mitochondrial distribution within the axon. Although mitochondria distributed homogeneously, Miro1^KO^ axons were shorter compared to WT (Figure 4C; p = 0.004, t test). As mitochondria were observed at the tip of the axon (approximately 650 μm from the soma) within both WT and Miro1^KO^ processes, the defects in axonal growth are not due to the lack of mitochondria in the most distal regions of the axon.

To test these observations in vivo, we analyzed the Stratum Oriens (SO) or Stratum Radiatum (SR) from the contralateral hippocampus of our AAV-injected mouse brains, which revealed that axonal mitochondrial distribution from the CA3 neurons transduced with GFP and MtdsRed2 (Figure 4D) is unaffected by Miro1 deletion even at 8 months of age (Figures 4E and 4F; number of mitochondria/10-μm axon: WT 2.31 ± 0.13, Miro1^CKO^ 2.18 ± 0.13; p = 0.505, t test), while changes in mitochondrial distribution in dendrites were evident much earlier, at 4 months. This analysis was performed in regions of axons up to several millimeters away from the somas in the contralateral hippocampus demonstrating that the distribution of mitochondria within axons and dendrites differentially rely on Miro1 function.

### Miro1 Deletion In Vivo Triggers Neurodegeneration

**Figure 5:**
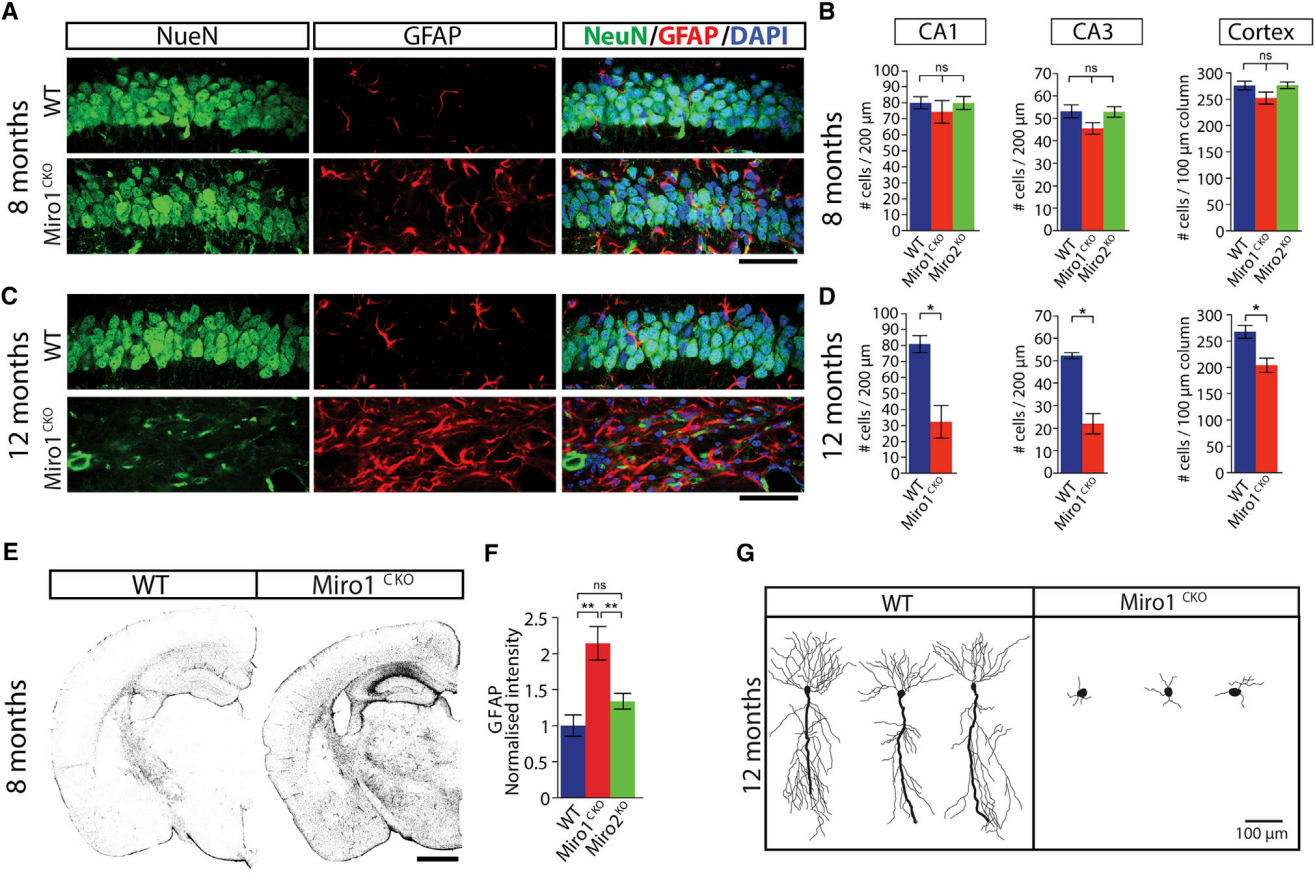
Miro1 Deletion in Mature Neurons Leads to Neurodegeneration (A and C) Images of the CA1 region of the hippocampus from WT and Miro1^CKO^ animals at 8 (A) and 12 (C) months of age. Neuronal marker NeuN (green), glial marker GFAP (red), and nuclear staining DAPI (blue) (scale bar, 50 μm). (B and D) Number of NeuN positive cells in 200-μm-width fields of CA1 and CA3 regions and 100 μm width columns from the somatosensory area of the cortex from WT, Miro1^CKO^, and Miro2^KO^ animals at 8 (B) and 12 (D) months. (E and F) Confocal images of hemibrains immunostained with GFAP (grayscale) of WT and Miro1^CKO^ animals at 8 months (E) (scale bar, 1 mm). Normalized GFAP intensity measured on hemibrains (F) at 8 months of age from WT, Miro1^CKO^, and Miro2^KO^ animals (n = animals, 8 months WT = 4, Miro1^CKO^ = 4, Miro2^KO^ = 4; ANOVA-NK). (G) Golgi-stained neurons from the CA1 region of WT and Miro1^CKO^ brains at 12 months of age. ^∗^p < 0.05, ^∗∗^p < 0.01, ^∗∗∗^p < 0.001. Error bars are SEM.

In Miro1^CKO^ animals at 8 months of age, when a significant reorganization of dendritic architecture and a decrease in cortex and hippocampal size is observed, there is no significant loss of neurons, detected by quantification of NeuN staining in these regions (Figures 5A, 5B, and S5F). However, the loss of dendritic complexity at this time point was accompanied by a 2-fold increase in GFAP signal (p < 0.01) and infiltration of reactive astrocytes, possibly as a protection mechanism to scavenge and clear cellular material product from the initial distal dendritic loss (Figures 5A, 5E, and 5F). At a later time point, 12 months, Miro1^CKO^ animals showed an enhanced loss of the dendritic tree in CA1 pyramidal neurons and a marked decrease in the number of neurons compared to WT brains (Figures 5C, 5D, 5G, and S5F).

Accordingly, the absence of Miro1-mediated mitochondrial positioning leads to a progressive dendritic loss that eventually results in neuronal degeneration.

## Discussion

Using constitutive and conditional mouse knockout strategies we demonstrate that Miro1 is the main regulator of mitochondrial transport and localization, important for ensuring correct dendritic development, maintenance, and neuronal survival. Miro1^KO^ neurons have less anterogradely and retrogradely moving mitochondria in both dendrites and axons, whereas knocking out Miro2 has no significant effect suggesting that either Miro1 can compensate for loss of Miro2 or that Miro2 has evolved other roles for mitochondrial function. Loss of Miro1 leads to accumulation of mitochondria in proximal dendrites, resulting in neurons with shorter dendrites but with significantly increased branching within proximal regions. Several earlier studies have supported links between mitochondrial function and neuronal development (Ishihara et al., 2009, Norkett et al., 2016, van Spronsen et al., 2013). Disrupting the mitochondrial fission and fusion machinery (e.g., by targeting mitofusins or Drp1) disrupts dendritic growth (Chen et al., 2007, Ishihara et al., 2009), but it is hard to separate effects on mitochondrial dynamics, function, and trafficking in these experiments, given that targeting the fission/fusion machinery can lead to depolarized mitochondria with altered genetic content (Chen et al., 2007, MacAskill and Kittler, 2010). By directly targeting the Miro trafficking machinery, we have avoided the potential non-mitochondrial effects of targeting the TRAKs (Birsa et al., 2013, Kimura and Murakami, 2014, van Spronsen et al., 2013), the kinesin/dynein complexes (Tanaka et al., 1998), or artificially clustering mitochondria with the Golgi (Fukumitsu et al., 2015), which also has a critical role in dendritic morphogenesis (Horton et al., 2005). Interestingly, a similar effect is seen in vivo upon conditional CaMKIIα-Cre-driven, postnatal deletion of Miro1 in hippocampus and cortex demonstrating that the relationship between mitochondrial positioning and dendritic branch distribution plays a direct role in sculpting and maintaining mature neuronal circuits.

Remarkably mitochondrial distribution into axons is preserved in cultured neurons that lack Miro1 despite a notable 65% decrease of fast axonal mitochondrial trafficking. This is in stark contrast to the significant depletion of mitochondria from distal dendrites (which have a similar microtubule polarization as axons [Baas et al., 1988]) suggesting that different mechanisms are involved in the localization of mitochondria within axons and dendrites independent on microtubule orientation. We show that Miro2 overexpression can, to a small extent, rescue axonal mitochondrial trafficking in Miro1^KO^ cultured neurons. The increase in Miro2 protein levels in Miro1^CKO^ hippocampus may compensate the lack of Miro1 in these neurons and could explain the slow progression of the neurodegeneration phenotype and/or why Miro1^CKO^ axons are still populated with mitochondria. Coupling of mitochondria to cytoskeletal transport pathways via a Miro1-independent mechanism may also still occur, for example via Miro2 or an unknown TRAK1 adaptor (van Spronsen et al., 2013) or through the mitochondrial motor Kif1b (Nangaku et al., 1994). The small amount of remaining mitochondria transported in Miro1^KO^ axons may be anchored by axonal syntaphilin (Kang et al., 2008) or by mitochondrial presynaptic capture (Courchet et al., 2013). The decreased motility of stabilized mitochondria in mutant axons may nonetheless impact mitochondrial dynamics and turnover resulting in mitochondria being less able to support axonal growth or branching (Spillane et al., 2013).

Conditional deletion of Miro1 by enolase2 promoter-driven Cre was recently proposed to cause axonal pathology in juvenile mice similar to an upper motor neuron disease (Nguyen et al., 2014). This genetic lesion is expected to affect multiple populations of neurons in brain, spinal cord, and peripheral nervous system starting as early in development as E13.5 (Kwon et al., 2006). In contrast, here we show that conditional deletion of Miro1 in mature neurons drives the loss of dendritic complexity, which is clearly evident 8 months after birth and is followed by neuronal loss (that is evident at 12 months).

A number of neurological and neurodegenerative diseases have been related to mitochondrial dysfunction and disruption of mitochondrial trafficking (Birsa et al., 2013, MacAskill and Kittler, 2010, Sheng and Cai, 2012), but the mechanisms underlying the pathophysiology of such disorders have frequently been difficult to identify. Axons are up to 10^4^ times longer than dendrites and have often been the focus of attention when studying neurological diseases associated with mutations in transport components (Millecamps and Julien, 2013). Our study cannot rule out an axonal component participating in the neurodegenerative phenotype observed in Miro1^CKO^ animals. However, our Rab7GFP trafficking experiments suggesting that trophic support is not affected and our analysis of mitochondrial distribution in axons from 8-month-old Miro1^CKO^ animals suggest that an axonal pathology is unlikely the origin of the degeneration phenotype. The idea that alterations in axonal transport (mitochondrial and other organelles) are either a necessary or sufficient causative factor in the neurodegenerative progression has recently been challenged, supporting the idea that axonal transport defects and axonal degeneration may course independently of each other (Marinkovic et al., 2012). It is well known, however, that the loss of dendritic complexity is a hallmark of a number of neurological and neurodegenerative diseases (Grutzendler et al., 2007, Kulkarni and Firestein, 2012). Furthermore, recent work in a mouse model of Alzheimer’s disease establishes a mechanistic link between dendritic structural degeneration and neuronal hyperexcitability resulting from changes in the electrical properties of the affected neurons and that may apply to other neurodegenerative diseases where changes in dendritic morphology occur (Šišková et al., 2014).

Altogether we demonstrate that Miro1 function is critical in regulating the development of the dendritic tree and in maintaining and sustaining arborization. Mature neurons lacking Miro1 inevitably lose their dendritic complexity and consequently their connectivity rendering them vulnerable to undergo a neurodegenerative process. With our work, we highlight the imperative need of a correct mitochondrial distribution in dendrites to sustain their architectural complexity and to protect against neurodegeneration. This pathomechanism may contribute to the neurodegenerative outcome of many human neurological diseases providing a cellular target of intervention for future therapeutic strategies.

## Experimental Procedures

### Stereotaxic Hippocampal Infection

Stereotaxic viral injections were performed on 3- and 7-month-old mice as described previously (MacAskill et al., 2014). Anesthetized animals were mounted on a stereotaxic apparatus, and the brain was exposed to 7 × 40-nl measures of AAV2-CAG-MtdsRed-ires-GFP (titer = 1.2 × 10^12^ genome copies [GC]/mL, UPenn Vector Core [Stephen et al., 2015]) injected at coordinates relative to Bregma (Medial/Lateral, Dorsal/Ventral, Rostral/Caudal; −1.4, −1.5 to −0.5, −1.8). Expression occurred in the injected brain region for approximately 4 weeks until tissues were obtained (Figure 4D). All experimental procedures were carried out in accordance with institutional animal welfare guidelines and licensed by the UK Home Office in accordance with the Animals (Scientific Procedures) Act 1986.

### Golgi Staining

Golgi staining of neurons was performed using the Rapid Golgi Stain Kit (FD NeuroTechnologies) following the manufacturer’s protocol. Neurons were imaged using a 20× objective and analyzed using Neurolucida software (MBF Bioscience).

### Image Acquisition and Analysis

#### Mitochondrial and Endosomal Trafficking

Mitochondria or Rab7GFP vesicles in neurons were live imaged with an Olympus BX60M upright microscope and 63× objective. Movies were processed in ImageJ, and transport parameters were obtained from the kymographs as previously described (Deinhardt et al., 2006, López-Doménech et al., 2012).

#### Sholl and Mitochondrial-Sholl Analysis

Confocal images were acquired on a Zeiss LSM700 upright confocal microscope using a 63× objective. Dendrite tracing and analysis were performed in Neuronstudio (Norkett et al., 2016). Mitochondrial-Sholl analysis was performed using a custom ImageJ plugin, which quantified the amount of MtdsRed2 pixels within shells radiating out from the soma at one-pixel intervals.

### Statistical Analysis

Excel software (Microsoft) and GraphPad Prism (GraphPad) were used to analyze the data. Student’s t test or Mann-Whitney test was used to test differences between two conditions. Comparison of multiple conditions with normally distributed data was performed by one-way ANOVA followed by post hoc Newman-Keuls test and for non-parametric data by Kruskal-Wallis test followed by post hoc Dunn’s correction. Statistical significance was fixed at p < 0.05, represented as ^∗^p < 0.05, ^∗∗^p < 0.01, and ^∗∗∗^p < 0.001. All values in text are given as average ± SEM. Error bars are SEM.

Additional details regarding animal strains, stereotaxic brain infections, neuronal culture and transfection, histology, immunoblotting, immunofluorescence, and image acquisition and analysis can be found in the Supplemental Experimental Procedures section.

## Author Contributions

G.L.-D., N.F.H., A.F.M., and J.T.K. designed the project and the experiments; G.L.-D., N.F.H., V.V., H.R., I.L.A.-C., and A.F.M. performed experiments. G.L.-D., N.F.H., and V.V. analyzed the data. G.L.-D., N.F.H., and J.T.K. wrote the manuscript.

## Supplemental Information

Document S1. Supplemental Experimental Procedures and Figures S1–S5

Document S2. Article plus Supplemental Information
