# Supplementary material for: Loss of Dendritic Complexity Precedes Neurodegeneration in a Mouse Model with Disrupted Mitochondrial Distribution in Mature Dendrites
Source: Cell Rep. 2016 Oct 4;17(2):317–27. doi: 10.1016/j.celrep.2016.09.004 (PMC5067282; doi:10.1016/j.celrep.2016.09.004)
Supplement: Document S1. Supplemental Experimental Procedures and Figures S1–S5 [file mmc1.pdf]

Cell Reports, Volume *17*

## Supplemental Information

# Loss of Dendritic Complexity Precedes Neurodegeneration in a Mouse Model with Disrupted Mitochondrial Distribution in Mature Dendrites

**Guillermo López-Doménech, Nathalie F. Higgs, Victoria Vaccaro, Hana Roš, I. Lorena Arancibia-Cárcamo, Andrew F. MacAskill, and Josef T. Kittler**

**Supplementary material**

**Supplementary experimental procedures**

## Animals

The *Rhot1* (MBTN_ EPD0066_2_F01; Allele: *Rhot1*$^{tm1a(EUCOMM)Wtsi}$) and *Rhot2* (MCSF_ EPD0389_5_A05; *Rhot2*$^{tm1(KOMP)Wtsi}$) mice lines were obtained from the Wellcome Trust Sanger Institute as part of the International Knockout Mouse Consortium (IKMC) (Skarnes et al., 2011). The *Rhot1* transgenic line was generated following the Knockout-First strategy on C57BL/6J-Tyr$^{cBrd}$ and subsequently backcrossed in C57BL/6N Taconic Denmark strain. The *Rhot2* transgenic line was generated as a reporter-tagged deletion on C57BL/6N Taconic USA background. CAMKIIα-CRE strain has been described previously (Mantamadiotis et al., 2002). Animals were maintained under controlled conditions (temperature 20 ± 2°C; 12 hour light-dark cycle). Food and water were provided *ad libitum*. The genotyping was carried out following Sanger's recommended procedures, briefly the DNA was extracted from ear biopsies and PCRs were performed with the following primers (5' to 3'): Rhot1_16_F: TTAGGATTTGTACTTTGCCCCTG; Rhot1_16_R: AAAACCCTTCCTGCATCACC; Rhot2_WT_F3: GGCGTTAGCTCCAGTGAGTC; Rhot2_WT_R3: AGGATGTGAAGAGGGTGGTG; Rhot2_mut_F4: GGGTTGAAGGAGAGGGTTG; CAS_R1_Term: TCGTGGTATCGTTATGCGCC; LacZ_2_small_F: ATCACGACGCGCTGTATC; LacZ_2_small_R: ACATCGGGCAAATAATATCG. To test for CRE recombination we designed specific primers flanking the loxP sites: Rhot1_Ex2G_F: GGAGTAGAGAAGTCAGATTCCAG and Rhot1_Ex2G_R: GAAGGCGTCAGATCACATTG. All experimental procedures were carried out in accordance with institutional animal welfare guidelines and licensed by the UK Home Office in accordance with the Animals (Scientific Procedures) Act 1986.

**Stereotaxic hippocampal infection**

Viral injections were performed on mice as described previously (Cetin et al., 2006; MacAskill et al., 2014). Stereotaxic injections were performed on mice at 3 and 7 months respectively, which were anaesthetized with ketamine (100 mg / kg) and xylazine (5 mg / kg) administered intraperitoneally (i.p.). Animals were gently mounted on a stereotaxic apparatus and a single incision was made along the midline to reveal the skull. The head was leveled and injection coordinates determined relative to bregma (Medial / Lateral, Dorsal / Ventral, Rostral / Caudal; BLA: -1.4, -1.5 to -0.5, -1.8). Long-shaft borosilicate pipettes with a tip diameter of 5 to 10 µm were backfilled with 0.8 µl of AAV2-CAG-MtdsRed-ires-GFP (titer = 1.2 x $10^{12}$ GC / ml, UPenn Vector Core, (Stephen et al., 2015)). A small hole was created in the skull over the injection site using a fine dental drill and 7 x 40 nl measures of virus were injected at 3 different points over a 1 mm range resulting in a final injection volume of no more than 300 nl. The pipette was left in place for an additional 5 min to minimize diffusion and then slowly removed. The wound was sutured and sealed and the mice monitored until recovery on a heat pad before returning to their home cage. Expression occurred in the injected brain region for approximately four weeks until tissues were obtained (Figure 4D).

**Cell culture and transfection**

Hippocampal cultures were obtained from E16 mouse brains from crosses of heterozygous Miro1 (or Miro2) animals. After dissecting each embryo a sample from the tail was used for genotyping and the hippocampi were further processed, separated and identified at all times. Cells were seed at a density of 30,000-50,000 cells/$cm^2$ onto Poly-L-Lysine (0.5 mg/ml in 0.1M borate buffer, pH 8) coated coverslips and transfected at 12 DIV by calcium phosphate or Lipofectamine2000 (Invitrogen). 30% of the medium was replaced with fresh medium every 3-4 days starting at 7DIV.

**DNAs, reagents and antibodies.**

GFP, MtdsRed2 and Miro1-myc have been previously described (Macaskill et al., 2009). Su9-EGFP was a gift from David Chan (Addgene plasmid # 23214) and pRK5-myc-Miro2 was a gift from Pontus Aspenström (Addgene plasmid # 47891), both obtained from addgene. FluoroNissl (NeuroTrace 520/525) was purchase from Mollecular Probes (Invitrogen) and used following recommended guidelines. For immunocytochemistry and immunohistochemistry antibodies were used as follows: rat anti-GFP (1:1000, Nacalai-Tesque, GF090R); mouse anti-AnkG (1:200, NeuroMab); rabbit anti-Miro1 (1:250, Atlas Antibodies); rabbit anti-Tom20 (1:300, SantaCruz); rabbit anti-GFAP (1:1000, Dako); mouse anti-NeuN (1:300, chemicon). For westernblotting antibodies were used as follows: rabbit anti-Miro1 (1:1000, Atlas Antibodies); mouse anti-Miro2 (1:1000, NeuroMab); rabbit anti-actin (1:10000, Sigma), mouse ApoTrak-cocktail (1:1000, MitoSciences). Horse radish peroxidase-conjugated anti-mouse or anti-rabbit IgGs where used at 1:10000 (Rockland).

**Western blotting and immunocytochemistry**

Hippocampal cultures where fixed when required in 4% PFA for 10 min at RT and rinsed several times in PBS. For immunocytochemistry coverslips where permeabilized in PBS with 0.1% Triton-X100 and incubated 1 hour in blocking solution (1% BSA, 10% fetal bovine serum, 0.2M glycine in PBS with 0.1%Triton-X100). Primary antibodies where applied in blocking solution at the desired concentration and incubated for 2 hours. AlexaFluor (Invitrogen) secondary antibodies where incubated for one hour at 1:800 in blocking solution. Coverslips were mounted in Mowiol mounting media and kept in the dark at 4°C until imaged. For western blotting 20 μg of protein from lysates of whole E18 forebrain, or adult cortex, hippocampus or cerebellum were loaded on 8-12% acrylamide gels and transferred to nitrocellulose membranes (GE Healthcare Bio-Sciences) using the Biorad system. Membranes where blocked in 3% BSA on TBS-T or 4% powder milk in PBS-T for 1-2 hours.

Antibodies were incubated in blocking solution (overnight at 4°C for primary antibodies or 1 hour at RT for HRP-conjugated secondary antibodies). Membranes were developed using the ECL-Plus reagent (GE Healthcare Bio-Sciences) and acquired in a chemiluminescence imager coupled to a CCD camera (ImageQuant LAS 4000mini). Densitometric analysis was performed using Quantity-One software (Biorad).

**Tissue processing**

Animals of the selected age were culled by cervical dislocation or $CO_2$ exposure. Hemibrains were either snap-frozen in liquid nitrogen for protein studies, fixed in chilled 4% PFA for further processing for histology studies or immersed in Golgi solution for dendritic morphometric analysis. Animals infected with AAV encoding GFP and MtdsRed2 were anesthetized with isoflurane and transcardially perfused with chilled 4% PFA to maintain mitochondrial integrity and further processed for histology analysis.

**Histology**

Hemibrains were fixed by immersion in 4% PFA overnight at 4°C, cryoprotected in 30% sucrose-PBS for 24-48 hours and stored at -80°C. Tissue was serially cryosectioned in a Bright OTF-AS Cryostat (Bright Instrument, Co. Ltd.) at 30 μm thickness and stored in cryoprotective solution (30% PEG, 30% glycerol in PBS) at -20°C until used. FluoroNissl (NeuroTrace 520/525) staining was performed following manufacturer instructions. Immunohistochemistry studies were performed in free-floating sections. Briefly, tissue was washed 3-5 times in PBS over 30 minutes and permeabilized in PBS-0.5% Triton-X100 over a total of 3-5 washes during 30 minutes. Sections were then blocked at RT in a solution containing 3% BSA, 10% fetal bovine serum and 0.2M glycine in PBS 0.5% Triton-X100 for 3-4 hours. Eventually, tissue was further blocked overnight at 4°C in the same blocking solution plus purified goat anti-mouse Fab-

fragment (Jackson Immunoresearch) at a concentration of 50 μg/ml to reduce endogenous background. Sections were then further washed and incubated overnight at 4°C in primary antibodies prepared in blocking solution. After washing in PBS 0.5% Triton-X100 secondary antibodies were applied in blocking solution and incubated at RT for 3-4 hours. Sections were mounted on glass slides using Mowiol mounting media and stored at 4°C in the dark until documented.

**Golgi Staining**

Golgi staining of neurons was performed using the Rapid Golgi Stain Kit (FD NeuroTechnologies) following the manufactures protocol. Neurons were imaged and analyzed using Neurolucida software (MBF Bioscience) attached to an upright light widefield microscope (Pathania et al., 2014). E18 brains were left in the impregnating solution for a maximum of 5 days only and adult brains were left for 2 weeks. Following impregnation brains were sliced at 100 μm thickness using a vibratome (Leica). Within E18 brains the processes of cortical neurons were imaged using a 60x objective (Olympus, NA 1.35) and at least 10 neurons traced per a brain. The dendritic morphology of CA1 hippocampal neurons of adult brains were imaged using a 20x (Olympus, NA 0.45) objective. The entire dendritic tree of at least three neurons was traced and averaged to produce one value per animal. All animals were age matched in each time-point. Miro1$^{lox/lox,Cre(-/-)}$ (WT controls) and Miro1$^{CKO}$ animals were obtained from the same litters from each individual experiment.

**Image acquisition and analysis**

*Mitochondrial and endosomal trafficking.* Neurons were live imaged with an Olympus BX60M upright microscope using a 63X water immersion objective (NA: 1.0) and a homemade setup to allow a constant flow of 36.5°C warmed ACSF (artificial cerebrospinal fluid; 10mM HEPES, 125mM NaCl, 10mM D-Glucose, 5mM KCl, 2mM

CaCl2 and 1mM MgCl2 at pH7.4). For synaptic stimulation experiments 30 μM glutamate and 1 μM glycine were included in the buffer and perfused in the cultures for one minute. Images were acquired using an EM-CCD camera (Ixon; Andor, Oxford Instruments). All electronics were controlled by AndorIQ Software (Oxford Instruments) or μManager (http://micro-manager.org/wiki/Micro-Manager). Proximal axonal segments situated at least 50 μm away from the soma and principal dendrites other than the primary dendrite were selected from the GFP channel and mitochondria were imaged on the MtdsRed2 channel. Images of selected dendrites or axons were acquired every 2 seconds for a total time of 2 minutes (61 frames in total). For Rab7GFP trafficking studies movies were generated at a rate of 1 image every second for a total of 2 minutes. Generated movies were processed with ImageJ software (http://imagej.nih.gov/ij/). Alignment (stackreg) and background subtraction were applied when required. Kymographs were generated from the selection traces obtained from the GFP image. Transport parameters were obtained from the kymographs as previously described (De Vos and Sheetz, 2007; Deinhardt et al., 2006; Lopez-Domenech et al., 2012). In stimulation experiments analysis was performed in the two minutes previous to the stimulation and in the two minutes that followed the end of the stimulation. In Miro1 experiments at DIV 14 all data was obtained from nine different preparations (five of which included Miro1-myc rescue experiments). Miro2 overexpression studies in Miro1$^{KO}$ neurons were conducted over five additional independent preparations. For the Miro2$^{KO}$ experiments four different preparations were used. All 6 DIV in vitro data was obtained from 5 different experiments.

*Confocal imaging.* Confocal images were acquired on a Zeiss LSM700 upright confocal microscope (Carl Zeiss, Welwyn Garden City, UK) using a 63X oil immersion objective (NA: 1.4). Images were stitched together using Vias (CNIC software tools) to allow the whole dendritic arbor of the neuron to be visualised. Dendrites were then

traced using Neuronstudio (Wearne et al., 2005) and the total dendritic length, number of branch points and Sholl analysis were automatically calculated. Sholl analysis to reveal the mitochondrial distribution was performed using an ImageJ plugin designed within the lab which quantified the amount of MtdsRed2 pixels within shells radiating out from the soma at 1 pixel intervals. For histology studies confocal images were taken using a 5X (NA: 0.16) or 10X (NA: 0.3) air objective or a 63X oil immersion objective (NA: 1.4). Acquisition parameters were kept constant over experimental conditions. All histology experiments were performed with age matched animals. Three different measures from consecutive brain slices (spanning 150 to 200 μm) were taken for each independent parameter (cortical thickness, number of neurons in each region measured or GFAP intensity in hemibrains) and averaged to produce one value per animal. For mitochondrial and endosomal trafficking assays MtdsRed2 and Rab5-GFP were transfected in hippocampal neurons and simultaneously imaged at 14 DIV using a 63X water immersion objective (NA: 1.0) and a constant flow of 36.5°C warmed ACSF. Frames were obtained every 2 seconds for a total of 2 minutes. For analysis of mitochondrial distribution in AAV infected brains segments of axons (in contralateral hippocampus) or dendrites (in ipsilateral hippocampus of the infection area) of at least 50 μm length were imaged with a 63X oil immersion objective. Two animals were used per age selected and genetic condition.

*Mitochondrial membrane potential in hippocampal neurons*. Dissociated cortical neurons were pooled together from all E16 embryos and seeded at a density of 40.000 cells/cm$^2$ to serve as a supporting culture. Hippocampal cells from individual embryos were then split into 2 different tubes and nucleofected (AMAXA Biotechnology) with either GFP or CFP as reporters. Combinations of differentially nucleofected (GFP and CFP) hippocampal neurons from 2 different embryos were then seeded at a density of 5.000 cells/cm$^2$ (each embryo) over the supporting culture and left 14 days to develop. After genotyping, the coverslips with cocultured neurons from a WT and a KO embryo

together were selected for the experiments. These coverslips were incubated for 30 minutes at 37°C with the mitochondrial membrane potential sensor tetramethylrhodamine methyl ester (TMRM, Biotium) at 20 nM and then live imaged on a Zeiss LSM700 upright confocal microscope (Carl Zeiss, Welwyn Garden City, UK) using a 20X water immersion objective (NA: 1.0) on a constant flow of 36.5°C warmed ACSF. Stack images were taken from fields that enclosed at least one GFP transfected neuron and one CFP transfected neuron. Fluorescent intensity was measured in the somas of the transfected cells from the maximum projection images (ImageJ, http://imagej.nih.gov/ij/) and then normalized with the average intensity of at least 3 non-trasfected neurons from the supporting culture. Normalized values for each condition were obtained from 3 different experiments.

## Supplementary Figure legends

### Supplementary Figure S1. Related to Figure 1:

(A) *Rhot1* transgenic allele ($Rhot1^{tm1a(EUCOMM)Wtsi}$) is a knockout-first (tm1a) with the insertion of an FRT flanked L1L2_gt1 cassette between exon 1 and 2 and LoxP sites flanking exon 2. Flp driven recombination of tm1a produces *Rhot1* conditional allele (tm1c). Miro1 expression is again possible, as En2 splice acceptor has been removed. This allele has the potential to become knockout when Cre recombination occurs producing the deletion allele (tm1d).

(B) *Rhot2* allele ($Rhot2^{tm1(KOMP)Wtsi}$) is of the type tagged deletion (tm1). Exons from 2 to 16 are substituted through homology recombination by an L1L2_Bact_PCassette cassette.

(C and D) PCR analysis on DNA extracted from E16 $WT^{(+/+)}$, $Het^{(+/-)}$ and $KO^{(-/-)}$ embryos shows successful recognition of either WT and *Rhot1* mutant allele (C) or *Rhot2* deletion allele (D).

(E) Western blot analysis of Miro1 and Miro2 protein levels from E18 brain lysates from litters generated by heterozygous mattings for the *Rhot1* allele (first 6 lanes) or *Rhot2* mattings (last 5 lanes). Antibody against Miro (Atlas) recognizes both Miro1 (duplex at ~71-73 KDa) and Miro2 proteins (~74 KDa).

(F-I) Mitochondrial stopping induced by glutamate stimulation in 15 DIV $Miro1^{CKO}$ neurons and in WT are undistinguishable.

(F) Kymographs of mitochondrial movement within $Miro2^{KO}$ neurons, dendrites (left) and axons (right).

(G) Merged kymographs showing simultaneous mitochondrial movement (red) and Rab5 positive endosomal movement (green) in WT and $Miro1^{KO}$ axons.

(L-M) Rab7 positive endosome dynamics within $Miro1^{KO}$ and WT. (L) Representative kymographs showing the dynamics of Rab7 positive endosomes within the axon. (M) Quantification of the percentage of moving vesicles (WT 46.6 ± 2.4, $Miro1^{CKO}$ 45.9 ±

1.1; p=0.78, t-test) and (N) their velocities shows no difference in the dynamics of Rab7 endosomes within WT and $Miro1^{KO}$ axons.

**Supplementary Figure S2. Related to Figure 2:**

(A-B).Analysis of mitochondrial transport in axons (A) and dendrites (B) in cultures at 14-15 DIV overexpressing Su9GFP or Miro2GFP. Transport analysis within the axon (A) indicates that Miro2 overexpression can only partially rescue the defects in mitochondrial transport due to the deletion of Miro1 (Data obtained from 4 independent experiments; ANOVA-NK)

(C) Mitochondrial probability map and (D) $Mito^{60}$ value (WT n=16, $Miro2^{KO}$ n=13, t-test, p=0.15), show no difference in the distribution of mitochondria within WT and $Miro2^{KO}$ dendrites.

(E-H) Quantification of dendritic morphology at 6 DIV. (E) Dendritic length, (F) number of branch points, (G) $BP^{90}$ Branch points and (H) sholl analysis of the number of branch points shows that there is no difference in dendritic complexity between WT and $Miro2^{KO}$ neurons. (WT n=17, $Miro2^{KO}$ n=12, t-test show no significance).

**Supplementary Figure S3. Related to Figure 1 and Figure 2:**

(A-F) Mitochondrial trafficking analysis of 6-7 DIV neurons in axons (A-C) and dendrites (D-E). As seen in 14-15 DIV Miro1$^{KO}$ neurons mitochondrial transport is disrupted

(G-H) At 6-7 DIV mitochondrial distribution is disrupted. (G) MPM curve and (H) the length normalised Mito60 value shows that mitochondria are accumulated towards proximal regions of neurons (WT= 12, Miro1$^{KO}$= 13, t-test p= 0.035). (I-L) No difference in the size of the dendritic arbors is seen in Miro1$^{KO}$ neurons at 6-7 DIV (dendritic length: WT 796 ± 59 μm, Miro1$^{KO}$ 760 ± 75 μm, p= 0.71; number of primary dendrites: 5.0 ± 0.40, Miro1$^{KO}$ 5.5 ± 0.37, p= 0.40; number of branch points: WT 13 ± 1.33, Miro1$^{KO}$ 13.8 ± 2.24, p= 0.73), however there are less branch points from 120 μm outwards from the soma, showing there is a decrease in dendritic complexity at this time-point (WT= 17, Miro1$^{KO}$= 18, t-test; p> 0.05, *p< 0.05)

(M) Representative image of a hippocampal neuron co-culture showing a WT neuron transfected with CFP (blue) and a Miro1$^{KO}$ neuron transfected GFP (green) labelled with TMRM (red).

(N) Quantification of mitochondrial membrane potential. Fluorescent signal from WT and Miro1$^{KO}$ neurons was normalized with an average measure of at least 3 neurons taken from the same image ("Ref" in graph). 30 images with one WT neuron and one Miro1$^{KO}$ neuron were used for the analysis. One value per condition was calculated in each experiment (n= 3). One-way ANOVA followed by Newman-Keuls *post hoc* correction for multiple comparisons was applied to test significant differences between groups.

**Supplementary Figure S4. Related to Figure 3 and Figure 5:**

(A) Western blot analysis from 4 month adult brains, probed for Miro1, Miro2 and actin protein. Dissected regions include cortex (Ctx), hippocampus (Hipp) and cerebellum (Cereb). As in E18 embryos the antibody against Miro1 (Atlas) recognizes both Miro1 (duplex at ~71-73 KDa) and Miro2 proteins (~74 KDa).

(B) Western blot of hippocampus lysates at different stages shows that Miro1 and 2 reactivity decreases during ageing during normal conditions. At 1 month of age Miro1 deletion is already significant and Miro2 appears to be upregulated.

(C) Difference in the size of brains dissected from WT vs Miro1$^{CKO}$ mice at 8 months.

(D-E) quantitative analysis of Miro2 levels in WT and Miro1CKO brains showing that Miro2 levels increase after Miro1 deletion.

**Supplementary Figure S5. Related to Figure 3 and Figure 5:**

(A) Analysis of the cortex width of Miro1$^{CKO}$ brains compared to WT and Miro2$^{KO}$ at 4 months (WT n= 3, Miro1$^{KO}$ n= 3, Miro2$^{KO}$ n= 3, Miro1/2$^{DKO}$ n= 4)

(B-E) Morphological analysis of 4 month old WT and Miro1$^{CKO}$ dendrites of pyramidal CA1 neurons showing that there is no difference in their dendritic morphology. (B) Reconstructed traces of CA1 neurons from 4 month old animals. Morphological quantification of the dendritic length (C) and the number of branch points (D) shows that at 4 months the dendritic architecture of Miro1$^{CKO}$ animals is indistinguishable from WT controls (n=animals, 4 months WT n= 3, Miro1$^{CKO}$ n= 3 and Miro2$^{KO}$ n= 3; ANOVA and *post hoc* Newman-Keuls). (E) Sholl analysis of the number of branch points shows that there is no difference in dendritic complexity.

(F) Number of NeuN positive cells counted in cortex and CA1 and CA3 regions of the hippocampus from the corresponding genotypes at 8 and 12 months of age.

*p<0.05, **p<0.01, ***p<0.001.

## Supplementary References


Cetin, A., Komai, S., Eliava, M., Seeburg, P.H., and Osten, P. (2006). Stereotaxic gene delivery in the rodent brain. Nature protocols *1*, 3166-3173.

De Vos, K.J., and Sheetz, M.P. (2007). Visualization and quantification of mitochondrial dynamics in living animal cells. Methods Cell Biol *80*, 627-682.

Deinhardt, K., Salinas, S., Verastegui, C., Watson, R., Worth, D., Hanrahan, S., Bucci, C., and Schiavo, G. (2006). Rab5 and Rab7 control endocytic sorting along the axonal retrograde transport pathway. Neuron *52*, 293-305.

Lopez-Domenech, G., Serrat, R., Mirra, S., D'Aniello, S., Somorjai, I., Abad, A., Vitureira, N., Garcia-Arumi, E., Alonso, M.T., Rodriguez-Prados, M., *et al.* (2012). The Eutherian Armcx genes regulate mitochondrial trafficking in neurons and interact with Miro and Trak2. Nature communications *3*, 814.

MacAskill, A.F., Cassel, J.M., and Carter, A.G. (2014). Cocaine exposure reorganizes cell type- and input-specific connectivity in the nucleus accumbens. Nat Neurosci *17*, 1198-1207.

Macaskill, A.F., Rinholm, J.E., Twelvetrees, A.E., Arancibia-Carcamo, I.L., Muir, J., Fransson, A., Aspenstrom, P., Attwell, D., and Kittler, J.T. (2009). Miro1 is a calcium sensor for glutamate receptor-dependent localization of mitochondria at synapses. Neuron *61*, 541-555.

Mantamadiotis, T., Lemberger, T., Bleckmann, S.C., Kern, H., Kretz, O., Martin Villalba, A., Tronche, F., Kellendonk, C., Gau, D., Kapfhammer, J., *et al.* (2002). Disruption of CREB function in brain leads to neurodegeneration. Nat Genet *31*, 47-54.

Pathania, M., Davenport, E.C., Muir, J., Sheehan, D.F., Lopez-Domenech, G., and Kittler, J.T. (2014). The autism and schizophrenia associated gene CYFIP1 is critical for the maintenance of dendritic complexity and the stabilization of mature spines. Translational psychiatry *4*, e374.

Skarnes, W.C., Rosen, B., West, A.P., Koutsourakis, M., Bushell, W., Iyer, V., Mujica, A.O., Thomas, M., Harrow, J., Cox, T., *et al.* (2011). A conditional knockout resource for the genome-wide study of mouse gene function. Nature *474*, 337-342.

Stephen, T.L., Higgs, N.F., Sheehan, D.F., Al Awabdh, S., Lopez-Domenech, G., Arancibia-Carcamo, I.L., and Kittler, J.T. (2015). Miro1 Regulates Activity-Driven Positioning of Mitochondria within Astrocytic Processes Apposed to Synapses to Regulate Intracellular Calcium Signaling. The Journal of neuroscience : the official journal of the Society for Neuroscience *35*, 15996-16011.

Wearne, S.L., Rodriguez, A., Ehlenberger, D.B., Rocher, A.B., Henderson, S.C., and Hof, P.R. (2005). New techniques for imaging, digitization and analysis of three-dimensional neural morphology on multiple scales. Neuroscience *136*, 661-680.

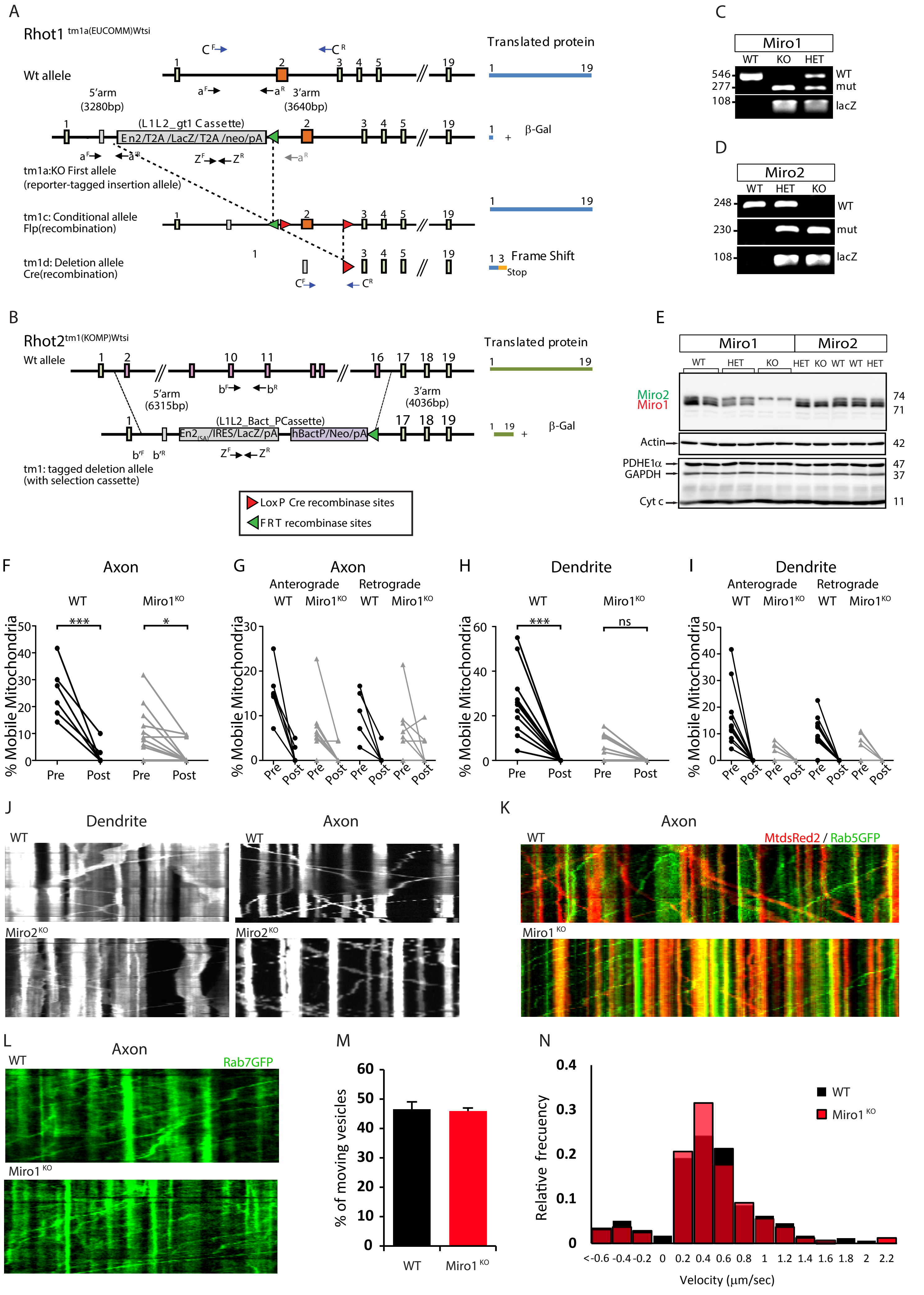


Supplementary Figure S1. Related to Figure 1

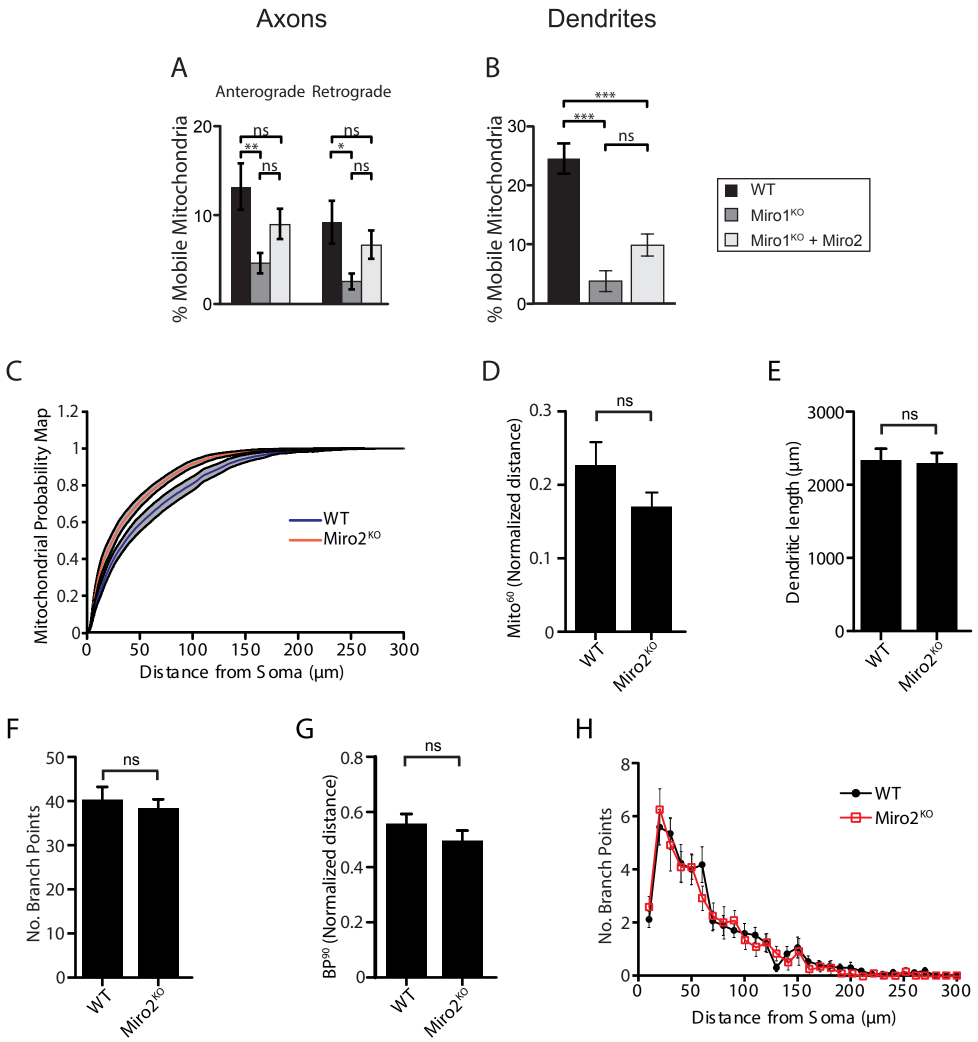


Supplementary Figure S2. Related to Figure 2

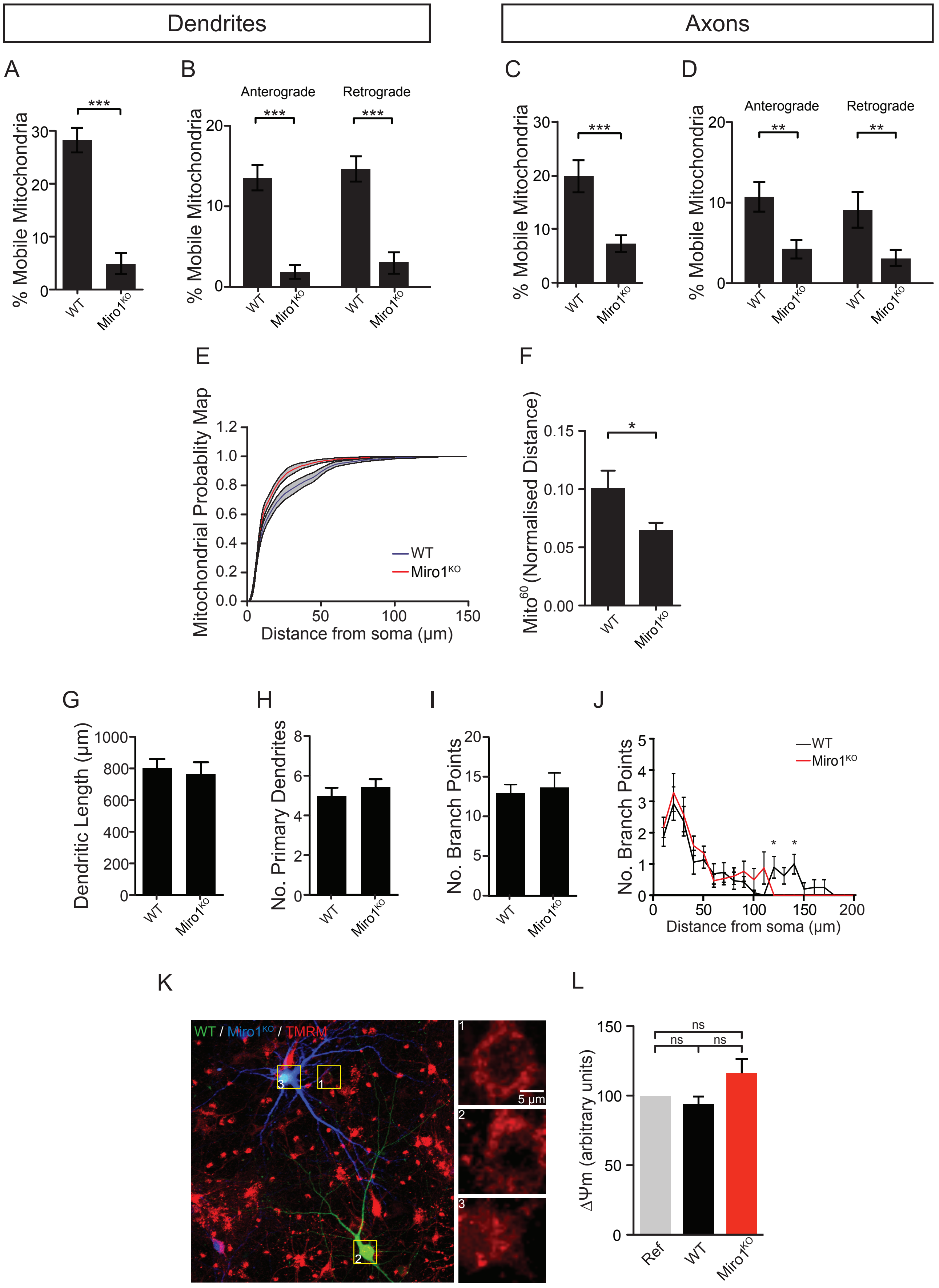


Supplementary Figure S3. Related to Figure 1 and Figure 2

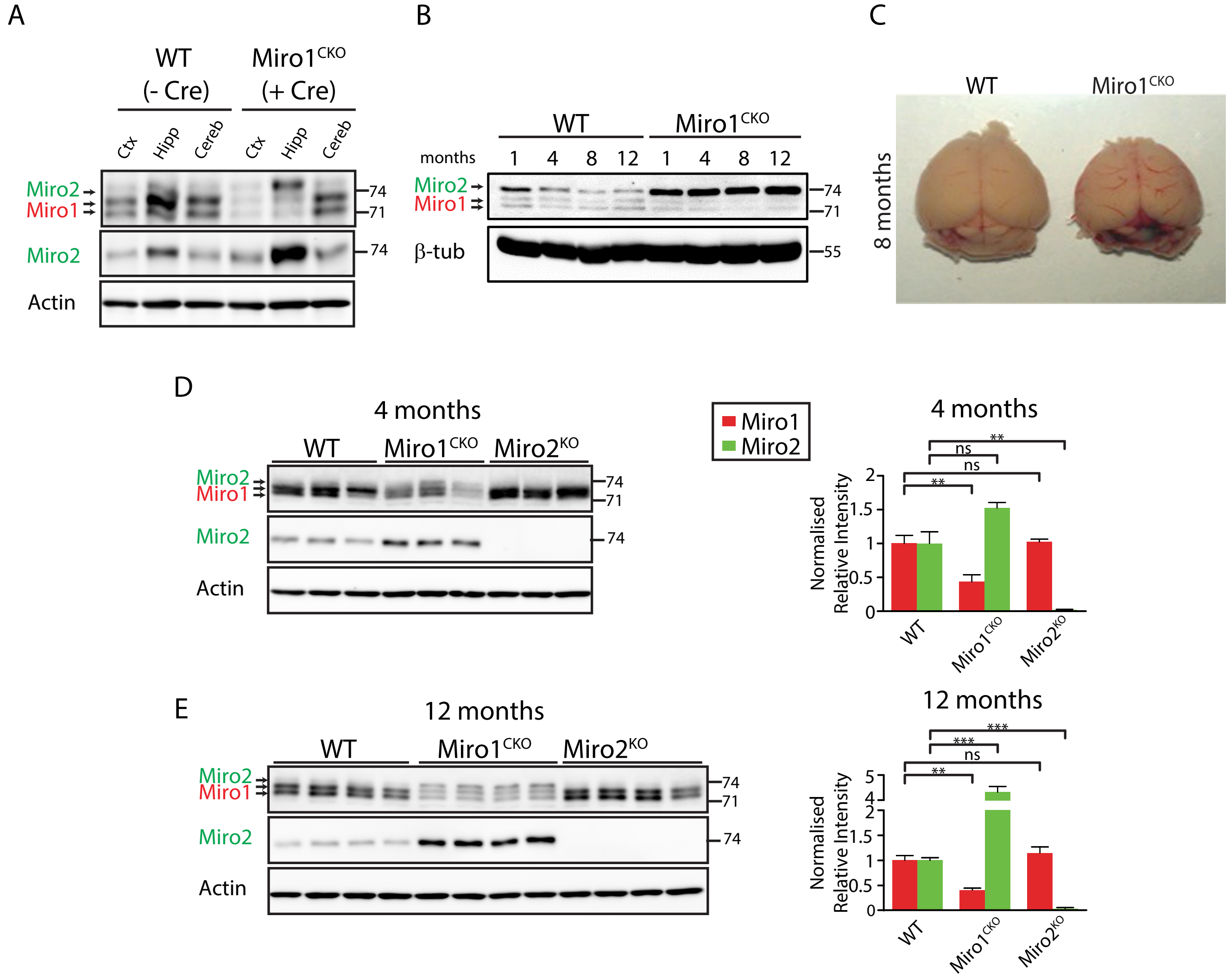


Supplementary Figure S4. Related to Figure 3 and Figure 5

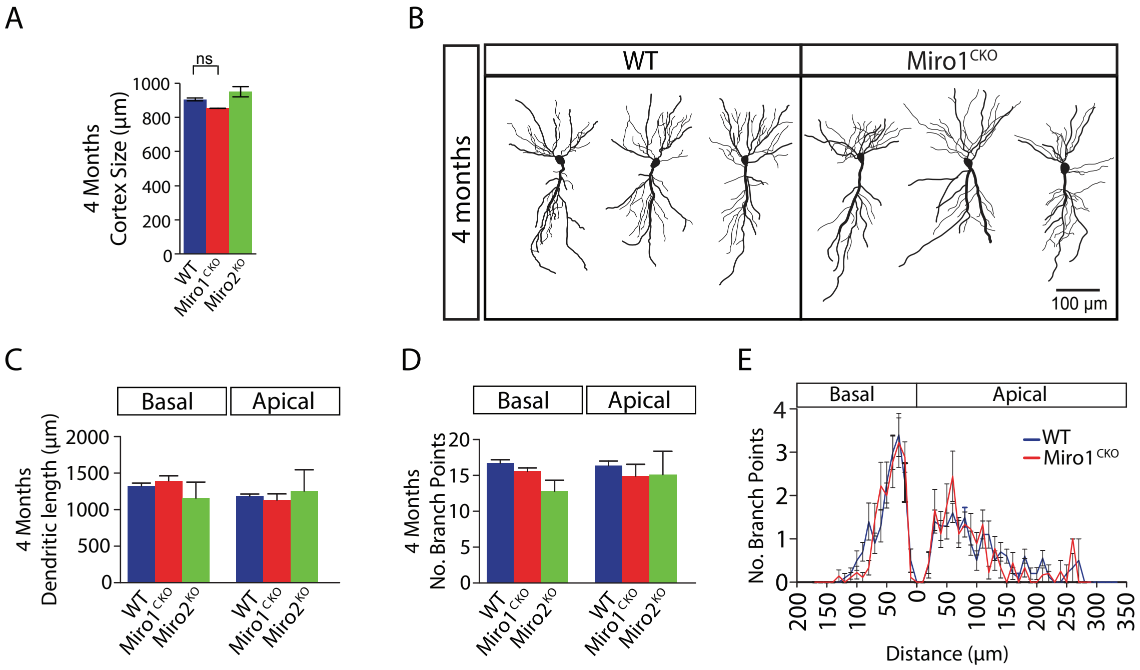


F

| Genotype | Cortex (cells in 100 mm columns) | CA1 (cells in 200 mm field) | CA3 (cells in 200 mm field) |
|---|---|---|---|
| **8 months** | | | |
| Wild type | 273.9 ± 7.9 | 79.8 ± 3.9 | 52.8 ± 2.8 |
| $Miro1^{CKO}$ | 250.3 ± 11.2 | 74.2 ± 6.9 | 45.3 ± 2.5 |
| $Miro2^{KO}$ | 274.4 ± 6.4 | 79.7 ± 4.2 | 52.6 ± 2.4 |
| **12 months** | | | |
| Wild type | 266.8 ± 12.5 | 80.7 ± 5.3 | 52.4 ± 1.3 |
| $Miro1^{CKO}$ | 203.7 ± 13.7 (*) | 32.3 ± 10.1 (*) | 22.1 ± 4.6 (*) |

Supplementary Figure S5. Related to Figure 3 and Figure 5
