# Supplementary material for: Loss of Dendritic Complexity Precedes Neurodegeneration in a Mouse Model with Disrupted Mitochondrial Distribution in Mature Dendrites
Source: Cell Rep. 2016 Oct 4;17(2):317–27. doi: 10.1016/j.celrep.2016.09.004 (PMC5067282; doi:10.1016/j.celrep.2016.09.004)
Supplement: Document S2. Article plus Supplemental Information [file mmc2.pdf]

Cell Reports

Report

Loss of Dendritic Complexity Precedes Neurodegeneration in a Mouse Model with Disrupted Mitochondrial Distribution in Mature Dendrites

Graphical Abstract

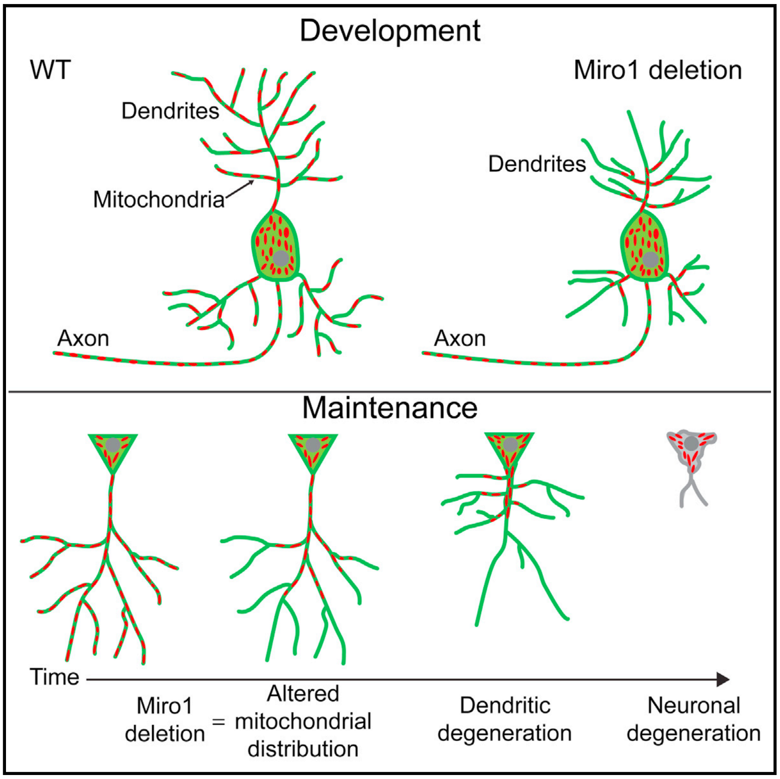

Authors

Guillermo López-Doménech, Nathalie F. Higgs, Victoria Vaccaro, Hana Roš, I. Lorena Arancibia-Cárcamo, Andrew F. MacAskill, Josef T. Kittler

Correspondence

j.kittler@ucl.ac.uk

In Brief

Complex dendritic morphologies are essential for neural circuit formation and brain computation. López-Doménech et al. demonstrate that Miro1-dependent dendritic mitochondrial positioning critically regulates the development of the dendritic tree and sustains arborization. Disrupting mitochondrial distribution in mature neurons leads to the loss of dendritic complexity, which precedes neurodegeneration.

Highlights

- Miro1 deletion alters mitochondrial distribution in dendrites but not axons
- Mitochondrial distribution impacts dendritic development
- Correct mitochondrial distribution is key to maintain complex dendritic geometries
- Aberrant dendritic mitochondrial distribution triggers neurodegeneration

López-Doménech et al., 2016, Cell Reports *17*, 317–327
October 4, 2016 © 2016 The Authors.
http://dx.doi.org/10.1016/j.celrep.2016.09.004

Cell Reports
Report

OPEN ACCESS
CellPress

Loss of Dendritic Complexity Precedes Neurodegeneration in a Mouse Model with Disrupted Mitochondrial Distribution in Mature Dendrites

Guillermo López-Doménech,[1,2] Nathalie F. Higgs,[1,2] Victoria Vaccaro,[1] Hana Roš,[1] I. Lorena Arancibia-Cárcamo,[1] Andrew F. MacAskill,[1] and Josef T. Kittler[1,3,*]
[1]Department of Neuroscience, Physiology and Pharmacology, University College London, Gower Street, London WC1E 6BT, UK
[2]Co-first author
[3]Lead Contact
*Correspondence: j.kittler@ucl.ac.uk
http://dx.doi.org/10.1016/j.celrep.2016.09.004

SUMMARY

Correct mitochondrial distribution is critical for satisfying local energy demands and calcium buffering requirements and supporting key cellular processes. The mitochondrially targeted proteins Miro1 and Miro2 are important components of the mitochondrial transport machinery, but their specific roles in neuronal development, maintenance, and survival remain poorly understood. Using mouse knockout strategies, we demonstrate that Miro1, as opposed to Miro2, is the primary regulator of mitochondrial transport in both axons and dendrites. Miro1 deletion leads to depletion of mitochondria from distal dendrites but not axons, accompanied by a marked reduction in dendritic complexity. Disrupting postnatal mitochondrial distribution in vivo by deleting Miro1 in mature neurons causes a progressive loss of distal dendrites and compromises neuronal survival. Thus, the local availability of mitochondrial mass is critical for generating and sustaining dendritic arbors, and disruption of mitochondrial distribution in mature neurons is associated with neurodegeneration.

INTRODUCTION

Although only 2% of the body's weight, the brain consumes 20% of the body's resting energy production (Harris et al., 2012), which may rise to 50% in the developing brain (Kuzawa et al., 2014). Energy in the adult brain is mainly used to reverse the ion influxes underlying synaptic and action potential signaling, for pumping neurotransmitters and for supporting signal transduction and trafficking events during synaptic plasticity (Harris et al., 2012; Rangaraju et al., 2014). During brain development energy may also be required to fuel the extensive neuronal growth necessary to build the complex neuronal morphologies (Kuzawa et al., 2014) that are essential for the formation of neuronal circuits (Jan and Jan, 2010) and for brain computation (Eyal et al., 2014; Spruston, 2008). While changes in dendritic complexity have been reported to occur in a number of neurological and neurodegenerative diseases or during normal aging (Grutzendler et al., 2007; Kabaso et al., 2009; Kulkarni and Firestein, 2012), the energy costs and cellular mechanisms underlying dendritic sustainability are poorly understood.

Mitochondria are critical for ATP provision and play other essential roles in neurons such as buffering calcium (MacAskill and Kittler, 2010; Sheng and Cai, 2012). The very large size of many neurons suggests that mitochondrial distribution must be spatially matched to local energy usage and calcium buffering requirements during the growth and maintenance of axons and dendrites. Indeed, alterations in mitochondrial dynamics compromise synaptic function (MacAskill and Kittler, 2010), and defective mitochondrial trafficking has been related to neurological and neurodegenerative disease (MacAskill et al., 2010; Sheng and Cai, 2012). Therefore, mitochondrial distribution and trafficking may be key determinants for both the generation and the long-term maintenance of the complex neuronal morphologies essential for brain information processing (MacAskill and Kittler, 2010; Sheng and Cai, 2012). Currently, however, the relationship between mitochondrial distribution and the morphogenesis and maintenance of neuronal architecture in vivo remains unclear.

Miro (mitochondrial Rho) GTPases are central regulators of mitochondrial trafficking that act as adaptors, linking mitochondria to motor proteins (Birsa et al., 2013; Fransson et al., 2006). Here, we use mouse genetics to investigate the differential role of Miro1 and Miro2 for mitochondrial trafficking, neuronal morphogenesis, and the maintenance of complex neuronal architecture. We show that Miro1 is the main regulator of mitochondrial trafficking and distribution. Depleting Miro1 levels in vivo during development disrupts neuronal morphogenesis, while postnatal Miro1 disruption in mature neurons leads to a loss of dendritic complexity starting in the distal dendrites. Our findings demonstrate that altering mitochondrial positioning in dendrites leads to a progressive loss of dendritic complexity, which, in turn, triggers neuronal death in a way that closely resembles a neurodegenerative process.

This is an open access article under the CC BY license (http://creativecommons.org/licenses/by/4.0/).

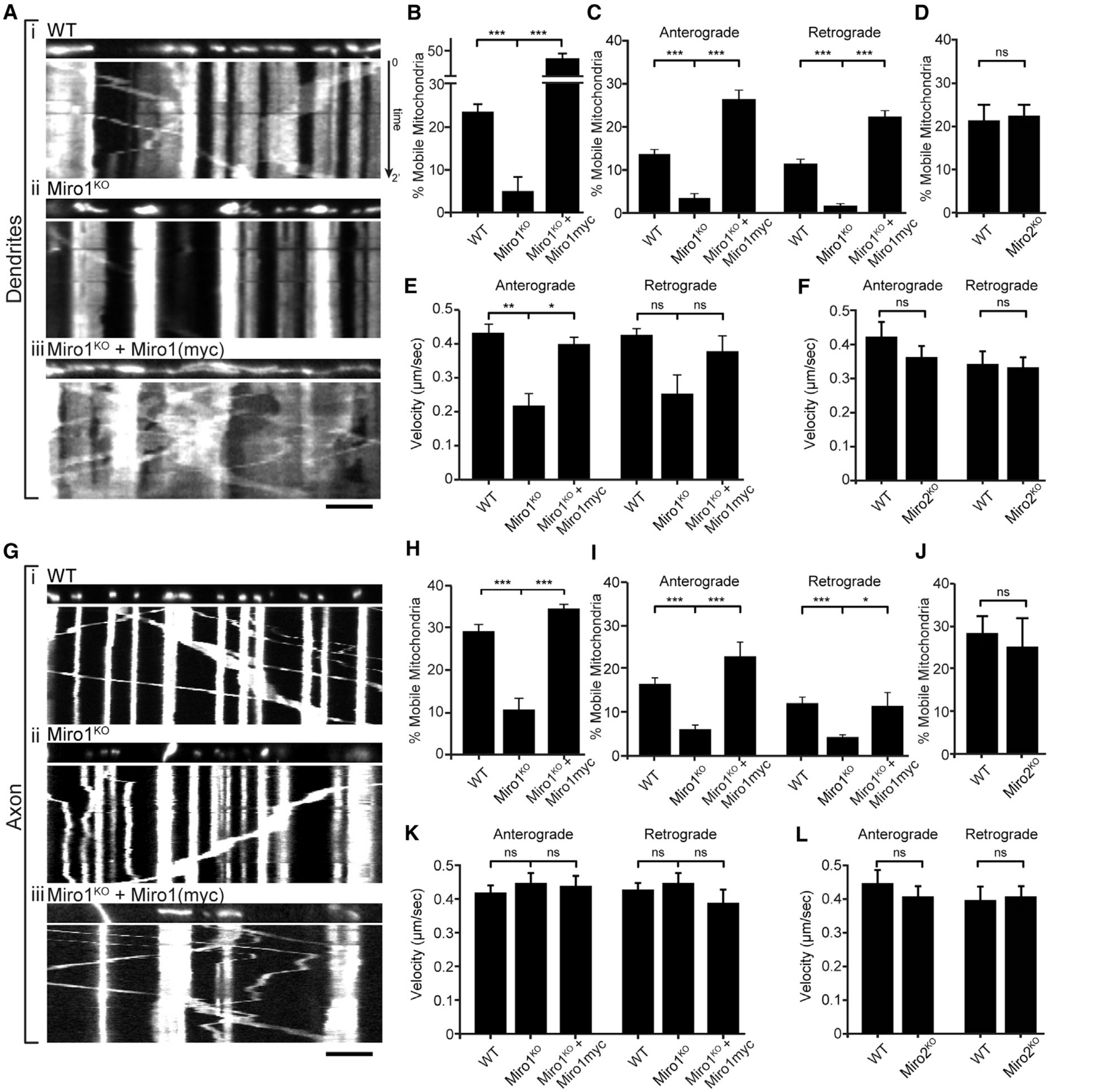

M

Genotype	Number of cells	% moving Mitochondria (%±SEM)	Anterograde (%±SEM)	Retrograde (%±SEM)	Number of mitochondria Ant	Number of mitochondria Retr	Anterograde velocity (µm/sec±SEM)	Retrograde velocity (µm/sec±SEM)
**Dendrites**								
Wild type (Miro1)	43	24.47±1.98	13.65±1.09	10.81±1.11	145	128	0.42±0.02	0.41±0.02
Miro1KO	23	4.76±1.38 (***)[a]	3.55±1.18 (***)[a]	1.20±0.52 (***)[a]	12	4	0.22±0.04 (**)[a]	0.25±0.11 (n.s.)[a]
Miro1KO + Miro1myc	25	47.00±3.14 (***)[b]	25.53±2.11 (***)[b]	21.47±1.55 (***)[b]	133	110	0.38±0.02 (*)[b]	0.36±0.02 (n.s)[b]
Wild type (Miro2)	12	21.24±3.56	11.74±2.17	9.50±1.61	29	23	0.42±0.04	0.34±0.04
Miro2KO	18	22.28±2.51 (n.s.)[c]	11.80±1.42 (n.s.)[c]	10.48±1.53 (n.s.)[c]	46	42	0.36±0.03 (n.s.)[c]	0.33±0.03 (n.s.)[c]
**Axons**								
Wild type (Miro1)	41	29.53±1.73	16.25±1.39	13.28±1.30	155	115	0.42±0.02	0.43±0.02
Miro1KO	33	10.47±1.11 (***)[a]	6.24±0.97 (***)[a]	4.23±0.60 (***)[a]	58	41	0.45±0.03 (n.s.)[a]	0.45±0.03 (n.s.)[a]
Miro1KO + Miro1myc	17	33.19±4.64 (***)[b]	21.22±2.90 (***)[b]	11.97±2.59 (*)[b]	69	52	0.44±0.03 (n.s.)[b]	0.39±0.04(n.s.)[b]
Wild type (Miro2)	9	28.34±3.99	16.63±3.34	11.71±2.24	33	26	0.45±0.04	0.40±0.04
Miro2KO	15	25.10±3.41 (n.s.)[c]	15.46±2.16 (n.s.)[c]	9.64±2.17 (n.s.)[c]	53	44	0.41±0.03 (n.s.)[c]	0.41±0.03 (n.s.)[c]

(legend on next page)

RESULTS

Miro1 and Miro2 Have Different Roles for Animal Viability and Mitochondrial Trafficking

To study the roles of Miro1 and Miro2, we characterized constitutive mouse knockout strains for *Rhot1* (Miro1 gene) and *Rhot2* (Miro2 gene) (Figures S1A–S1D) (Skarnes et al., 2011). Protein levels within brain lysates confirmed the specific deletion of Miro1 and Miro2 proteins, respectively (Figure S1E). *Rhot2* knockout animals (Miro2^{KO} hereafter) were viable and fertile, whereas *Rhot1* knockout animals (Miro1^{KO}) were born alive at the expected Mendelian ratios but remained cyanotic and died within the first 15 to 30 min of life (Nguyen et al., 2014).

To address the specific roles of Miro1 and Miro2 for mitochondrial trafficking, we compared hippocampal neuronal cultures from individual wild-type (WT) or knockout E16 embryos generated by heterozygous (Miro1$^{+/-}$ X Miro1$^{+/-}$ or Miro2$^{+/-}$ X Miro2$^{+/-}$) matings. In both 6–7 days in vitro (DIV) and 14–15 DIV Miro1^{KO} neurons expressing GFP to fill the cell and MtdsRed2 to label mitochondria, anterograde and retrograde mitochondrial trafficking was altered in dendrites (~85% decrease) and in axons (~65% decrease) (Figures 1 and S3A–S3D). The velocity of the remaining motile mitochondria at 14–15 DIV was unaltered in axons but was reduced by ~50% in the anterograde direction within dendrites whereas retrograde velocity was unaffected (Figures 1E, 1K, and 1M). Interestingly, the small amount of mitochondrial trafficking that remained was still sensitive to neuronal activation induced by glutamate (Macaskill et al., 2009) indicating that other mechanisms exist that can sense Ca^{2+} and induce Miro1-independent mitochondrial stopping (Figures S1F–S1I). Mitochondrial trafficking was fully rescued by expression of Miro1-myc (Figure 1) but only partially by Miro2 expression (Figures S2A and S2B). Unexpectedly, Miro2 deletion (Miro2^{KO} neurons) had no substantial effect on mitochondrial trafficking (Figures 1 and S1J) suggesting Miro2 is not the main regulator of mitochondrial trafficking or that its function can be compensated by Miro1. Moreover, the trafficking of Rab5GFP positive early endosomes (Figure S1K) and axonal retrograde transport of Rab7GFP positive signaling endosomes (Deinhardt et al., 2006) was unaffected in Miro1^{KO} neurons (Figures S1L–S1N) further confirming the critical importance and specificity of Miro1 deletion for mitochondrial trafficking.

Disruption of Dendritic Mitochondrial Distribution upon Miro1 Deletion

We found that in Miro1^{KO} neurons the vast majority of mitochondria were accumulated in proximal regions of dendrites and only sparsely distributed within distal dendrites, with large dendritic segments almost entirely devoid of mitochondria (Figures 2A and 2B). To quantify this effect, we performed a mitochondrial "Sholl" distribution analysis (see Experimental Procedures), to give a "mitochondrial probability map" (MPM): the cumulative probability plot of mitochondrial distribution as a function of distance from the soma. The MPM shows a clear shift to the left in the curve for Miro1^{KO} neurons at both 6 DIV and 14–15 DIV, confirming an accumulation of mitochondria in the proximal dendrites of Miro1^{KO} cells and reduction in distal dendrites (Figures 2C and S3E). To account for variability in neuronal process length, we defined a normalized value, Mito^{60}, the point at which 60% of the total mitochondrial mass is found within a normalized proximal to distal dendritic axis (where the dendritic tip is defined as 1). In Miro1^{KO} neurons the Mito^{60} value was decreased by ~40% at both 14–15 DIV ($p = 0.003$) and 6–7 DIV ($p = 0.035$), confirming a more accumulated distribution of mitochondria within proximal dendrites (Figures 2D and S3F). In contrast, in Miro2^{KO} neurons the MPM and Mito^{60} values were unaltered (Figures S2C and S2D, $p = 0.16$). Importantly, mitochondria accumulated in proximal Miro1^{KO} dendrites were metabolically active and kept their mitochondrial membrane potential, as revealed by tetramethylrhodamine methyl ester (TMRM) staining (Figures S3K and S3L). Thus, Miro1 control of mitochondrial trafficking is a key determinant of correct mitochondrial distribution within dendrites—a function that cannot be readily compensated by endogenous Miro2.

Miro1-Dependent Mitochondrial Trafficking and Distribution Are Critical for Correct Dendritic Development In Vitro and In Vivo

Neurons lacking Miro1 appeared smaller and less developed than control neurons at 14–15 DIV (Figure 2A). Total dendritic length was ~35% decreased in Miro1^{KO} neurons (Figure 2E, WT 3793 ± 89 μm, Miro1^{KO} 2447 ± 54 μm, $p = 0.0003$), although surprisingly the total number of branchpoints was unaffected (Figure 2F, WT 55.7 ± 2.78, Miro1^{KO} 57.9 ± 3.53, $p = 0.63$). Dendritic Sholl analysis of Miro1^{KO} neurons also revealed a marked increase in dendritic complexity in proximal regions and a decrease distally, closely correlating with the observed changes

Figure 1. Miro1, Not Miro2, Is the Main Regulator of Mitochondrial Transport

(A and G) Images and kymographs from dendrites (A) and axons (G) of hippocampal neurons from (1) WT, (2) Miro1^{KO}, and (3) with Miro1-myc rescue in Miro1^{KO} cultures. Images show mitochondria at time = 0 and corresponding kymographs show their motility over a 2-min period (height). Scale bar, 10 μm.

(B and H) Percentage of mobile mitochondria in dendrites (B) and axons (H) (dendrites WT = 43, Miro1^{KO} = 23, Miro1^{KO} + Miro1myc = 17, axons WT = 41, Miro1^{KO} = 33, Miro1^{KO} + Miro1myc = 15).

(C and I) Percentage of mitochondria moving in the anterograde or retrograde direction in dendrites (C) and axons (I) of WT, Miro1^{KO}, and Miro1^{KO} + Miro1myc neurons.

(D and J) Percentage of mobile mitochondria in WT and Miro2^{KO} dendrites (D) and axons (J) (dendrites WT = 12, Miro2^{KO} = 18, axons WT = 9, Miro2^{KO} = 15).

(E, F, K, and L) Average velocity of moving mitochondria from Miro1 (E and K) or Miro2 (F and L) experiments (dendritic mitochondria in (E): WT = 273, Miro1^{KO} = 16, Miro1^{KO} + Miro1myc = 243, and in (F) WT = 52, Miro2^{KO} = 88); (axonal mitochondria in (K) WT = 270, Miro1^{KO} = 101, Miro1^{KO} + Miro1myc = 121, and in (L) WT = 59, Miro2^{KO} = 97).

(M) Mitochondrial trafficking measured in 14-DIV hippocampal cultured neurons: (^{a}) compared to wild-type (Miro1 experiments); (^{b}) compared to Miro1^{KO} (Miro1 experiments); (^{c}) compared to wild-type (Miro2 experiments). Statistical differences were calculated assuming non-parametric distributions.

*$p < 0.05$, **$p < 0.01$, and ***$p < 0.001$. Error bars are SEM.

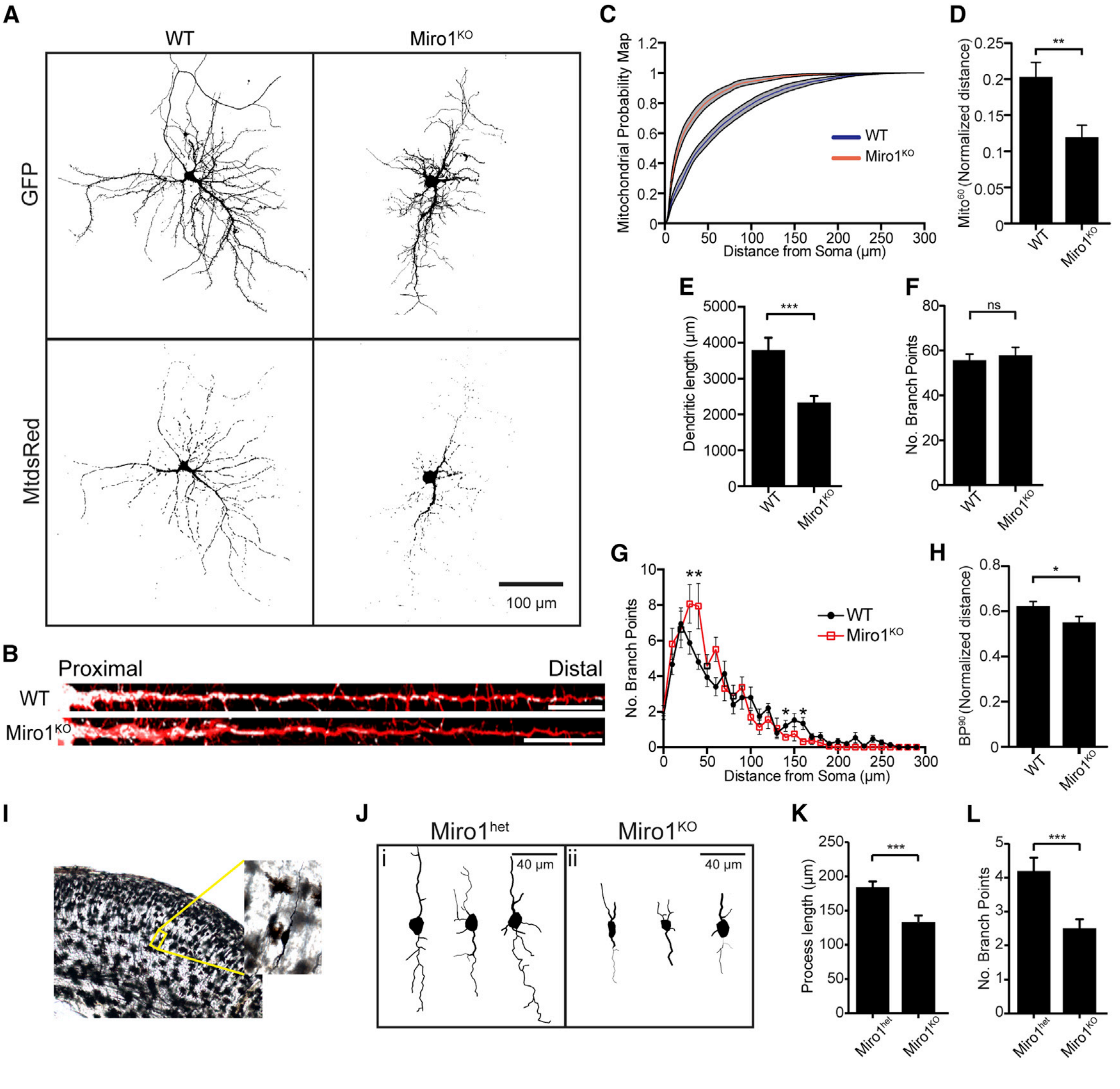

Figure 2. Miro1-Dependent Mitochondrial Distribution Drives Dendritic Morphogenesis and Arborization

(A) WT and Miro1^{KO} neurons expressing GFP and MtdsRed2 and imaged at 14 DIV (scale bar, 100 μm).

(B) Scaled dendritic process (red) showing mitochondrial distribution (white) in WT and Miro1^{KO} dendrites (scale bar, 30 μm).

(C and D) Mitochondrial probability map (MPM) (C) and the length-normalized Mito^{60} value from WT and Miro1^{KO} neurons (D) (number of cells: WT = 11 and Miro1^{KO} = 17).

(E–H) (E) Dendritic length and (F) number of branchpoints from WT and Miro1^{KO} neurons. Sholl analysis of branchpoint number (G) and the length-normalized BP^{90} value (H) (number of cells: WT = 15 and Miro1^{KO} = 19).

(I) Golgi staining of E18 brains and example traces (J) of E18 cortical neurons from Miro1^{KO} and Miro1^{het} control littermates. Process length (K) and number of branchpoints (L) from the traced neurons (n = 35 cells for each genotype).

*p < 0.05, **p < 0.01, ***p < 0.001. Error bars are SEM.

in mitochondrial distribution (Figure 2G). To account for changes in neuronal size, we defined a value that represented where 90% of the branchpoints are found within size-normalized cells (BP^{90}). The BP^{90} for Miro1^{KO} neurons is significantly less than for WT neurons as Miro1^{KO} neurons have significantly more branchpoints near the soma (Figure 2H). In contrast Miro2^{KO} neurons appeared similar to WT cells in terms of dendritic architecture (Figures S2E–S2H; dendritic length, p = 0.85; total branchpoints, p = 0.62) indicating that in the presence of Miro1, Miro2 is not required for correct dendritic development. Younger Miro1^{KO} neurons (6–7 DIV) were similar in terms of dendritic length and number of primary dendrites and branchpoints compared to WT (Figures S3G–S3I). Interestingly, Sholl analysis demonstrated that they were already beginning to show less distal complexity (120 μm outward from the soma) compared to WT neurons (Figure S3J). Moreover, reconstructions of Golgi-stained neurons (Figure 2I) located in the lower levels of the cortical plate of Miro1^{KO} brains showed a clear reduction in neurite length (p = 0.0009) and branchpoint number (p = 0.0005) (Figures 2J–2L) at an early stage of cortex development (at E18,

when Miro1^{KO} animals are viable) supporting a role for Miro1 in regulating neuronal morphogenesis in vivo. Thus local availability of mitochondrial mass is essential for correct neuronal process development.

Miro1 Is Necessary for Maintaining Mitochondrial Distribution and Dendritic Complexity in Mature Neurons In Vivo

To test whether the loss of Miro1 altered mitochondrial distribution in vivo and impacts postnatal dendritic maintenance, we generated a conditional Miro1 allele (Miro1$^{lox/lox}$, see Figure S1A) and crossed this with a strain expressing Cre recombinase under the Ca^{2+}/calmodulin-dependent protein kinase-alpha specific promoter (CaMKIIα-Cre), which has been widely used to drive Cre-mediated genetic deletion in postnatal forebrain. Miro1$^{\Delta/\Delta,Cre(+/-)}$ animals (hereafter Miro1^{CKO}) developed normally and appeared indistinguishable from their Miro1$^{lox/lox,Cre(-/-)}$ (WT hereafter) control littermates. Specific deletion of the Miro1 gene in Miro1^{CKO} animals occurred primarily in adult principal neurons of the cortex and hippocampus (Figure S4A). Interestingly, whereas, in WT animals Miro2 levels were progressively decreased over time (Figure S4B), this was not observed in brains deleted for Miro1 (Figures S4D and S4E) suggesting that Miro2 may be upregulated to counteract the depletion of Miro1.

By immunostaining with the mitochondrial marker Tom20 we observed in 4-month-old Miro1^{CKO} brains a decrease in the mitochondrial index (mitochondrial area/MAP2 positive dendritic area) in distal regions of the CA1 neurons corresponding to the Stratum Lacunosum-Moleculare (SLM), compared to WT (Figures 3A and 3B, $p = 0.043$; t test). At 8 months, Tom20 staining revealed an even more enhanced loss of mitochondrial density in Miro1^{CKO} CA1 distal dendrites (Figures 3A and 3B; $p < 0.001$; t test). To ensure that the loss in mitochondrial density was specifically in the dendrites and not in the axonal projections that populate the SLM region, we performed intracerebral injections of non-replicating Adeno-Associated Virus (AAV2) encoding MtDsRed2 and GFP (Stephen et al., 2015) into the CA1-CA3 region of the hippocampus. Neurons from control animals showed mitochondria distributed homogeneously throughout the dendritic length, whereas Miro1^{CKO} neurons presented long segments of distal dendrites devoid of mitochondria (Figures 3C and 3D, percentage of dendritic length without mitochondria; WT 25% ± 2%, Miro1^{CKO} 65% ± 5%; $p < 0.001$, t test), indicating that, also in vivo, Miro1 regulates mitochondrial distribution in mature dendrites.

Although dendritic mitochondrial distribution was already affected at 4 months of age, no defects in dendritic complexity were apparent when comparing Golgi-stained CA1 pyramidal neurons from WT and Miro1^{CKO} brains (Figures S5B–S5E: dendritic length: basal; WT 1321 ± 42 μm, Miro1^{CKO} 1394 ± 69 μm, apical; WT 1183 ± 31 μm, Miro1^{CKO} 1129 ± 86 μm; branchpoints: basal; WT 16.7 ± 0.5, Miro1^{CKO} 15.6 ± 0.8, apical; WT 16.3 ± 0.7, Miro1^{CKO} 14.8 ± 1.1; ANOVA and post hoc Newman-Keuls [ANOVA-NK]). In contrast, at 8 months, Miro1^{CKO} CA1 neurons showed a significant decrease in total dendritic length in both the basal and apical dendrites, (Figures 3E and 3G, basal: WT 1307 ± 100 μm, Miro1^{CKO} 889 ± 83 μm, $p < 0.01$; apical: WT 1686 ± 183 μm, Miro1^{CKO} 1155 ± 172 μm, $p < 0.05$; ANOVA-NK). Interestingly, while the number of branchpoints was similar to that of WT neurons (Figure 3H), Sholl analysis revealed more branchpoints in the proximal region of the apical dendrite and reduced branching in the distal regions in Miro1^{CKO} neurons (Figures 3F, 3I, and 3J). Age-matched Miro2^{KO} neurons showed no morphological defects (Figures 3G and 3H). The loss of dendritic complexity at 8 months in Miro1^{CKO} animals correlated with a clear decrease in brain size compared to WT (Figure S4C) and an increase in the size of the ventricles with a reduction in hippocampal size (Figures 3K and 3L, pial to apical surface distance of hippocampus: WT 516.8 ± 9.7 μm, Miro1^{CKO} 363.3 ± 26.7 μm, $p < 0.01$; and Miro2^{KO} 517.8 ± 14.9 μm, ANOVA-NK) and thickness of the cortex (Figures 3K and 3M, WT 960.4 ± 35.1 μm, Miro1^{CKO} 824.3 ± 24.4 μm, $p < 0.05$; and Miro2^{KO} 946.1 ± 35.4 μm, ANOVA-NK).

Therefore, the postnatal loss of Miro1 disrupts mitochondrial distribution in the proximal to distal dendritic axis leading to a gradual decrease in dendritic complexity particularly affecting the most distal dendrites.

The Distribution of Mitochondria along the Axon Is Not Dependent on Miro1

As opposed to dendrites, mitochondria distributed homogeneously throughout axons of Miro1^{KO} cultured hippocampal neurons (Figure 4A). A plot of the relative mitochondrial positions along WT and Miro1^{KO} axons from 6-DIV neurons, transfected with GFP and MtdsRed2 and normalized for changes in length (Figure 4B), showed that mitochondria are distributed throughout the entire length of the axon, despite the fact that fast axonal mitochondrial trafficking is severely altered in Miro1^{KO} neurons. This suggests that other mechanisms can compensate the absence of Miro1 for mitochondrial distribution within the axon. Although mitochondria distributed homogeneously, Miro1^{KO} axons were shorter compared to WT (Figure 4C; $p = 0.004$, t test). As mitochondria were observed at the tip of the axon (approximately 650 μm from the soma) within both WT and Miro1^{KO} processes, the defects in axonal growth are not due to the lack of mitochondria in the most distal regions of the axon.

To test these observations in vivo, we analyzed the Stratum Oriens (SO) or Stratum Radiatum (SR) from the contralateral hippocampus of our AAV-injected mouse brains, which revealed that axonal mitochondrial distribution from the CA3 neurons transduced with GFP and MtdsRed2 (Figure 4D) is unaffected by Miro1 deletion even at 8 months of age (Figures 4E and 4F; number of mitochondria/10-μm axon: WT 2.31 ± 0.13, Miro1^{CKO} 2.18 ± 0.13; $p = 0.505$, t test), while changes in mitochondrial distribution in dendrites were evident much earlier, at 4 months. This analysis was performed in regions of axons up to several millimeters away from the somas in the contralateral hippocampus demonstrating that the distribution of mitochondria within axons and dendrites differentially rely on Miro1 function.

Miro1 Deletion In Vivo Triggers Neurodegeneration

In Miro1^{CKO} animals at 8 months of age, when a significant reorganization of dendritic architecture and a decrease in cortex and hippocampal size is observed, there is no significant loss

OPEN
ACCESS
CellPress

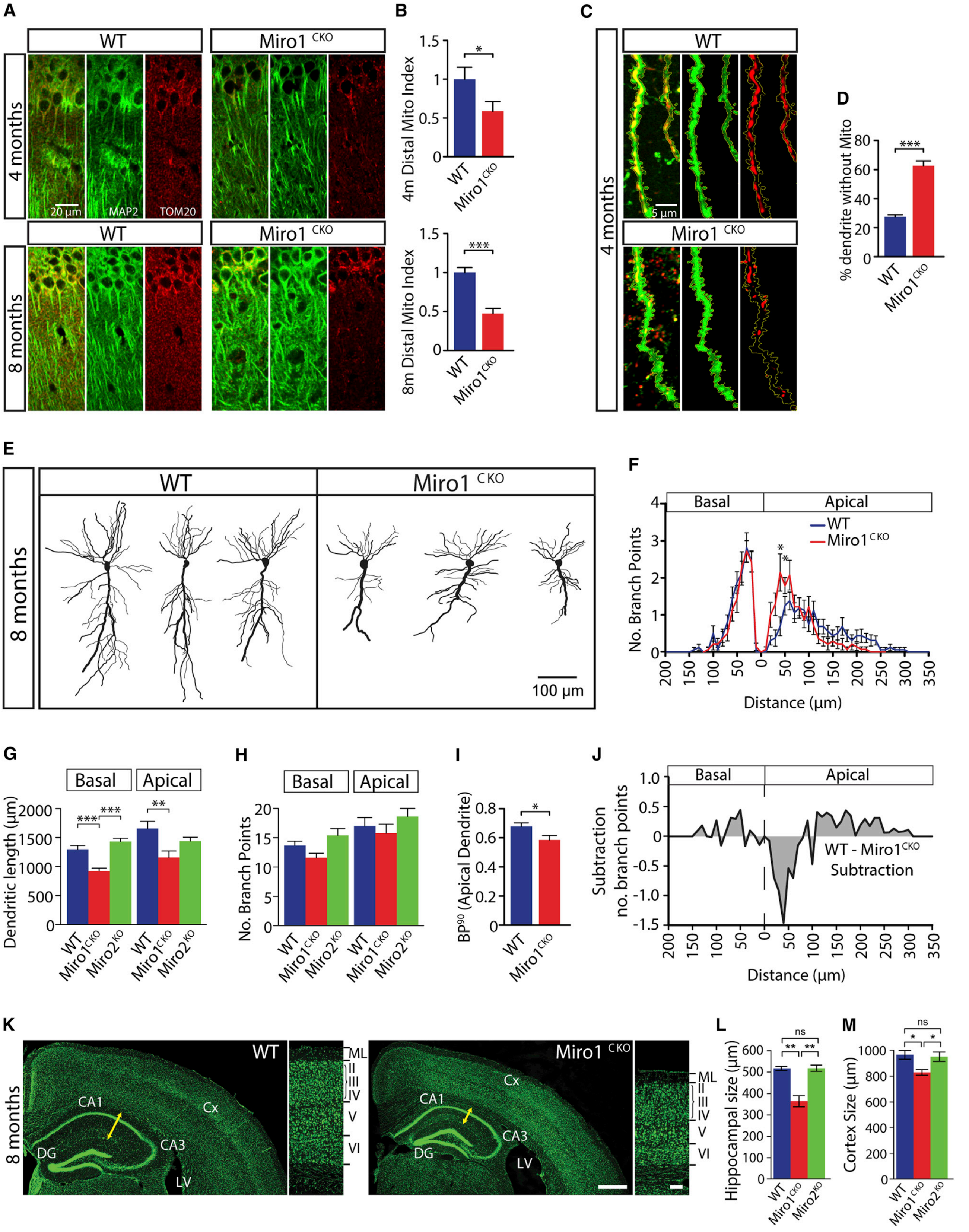

(legend on next page)

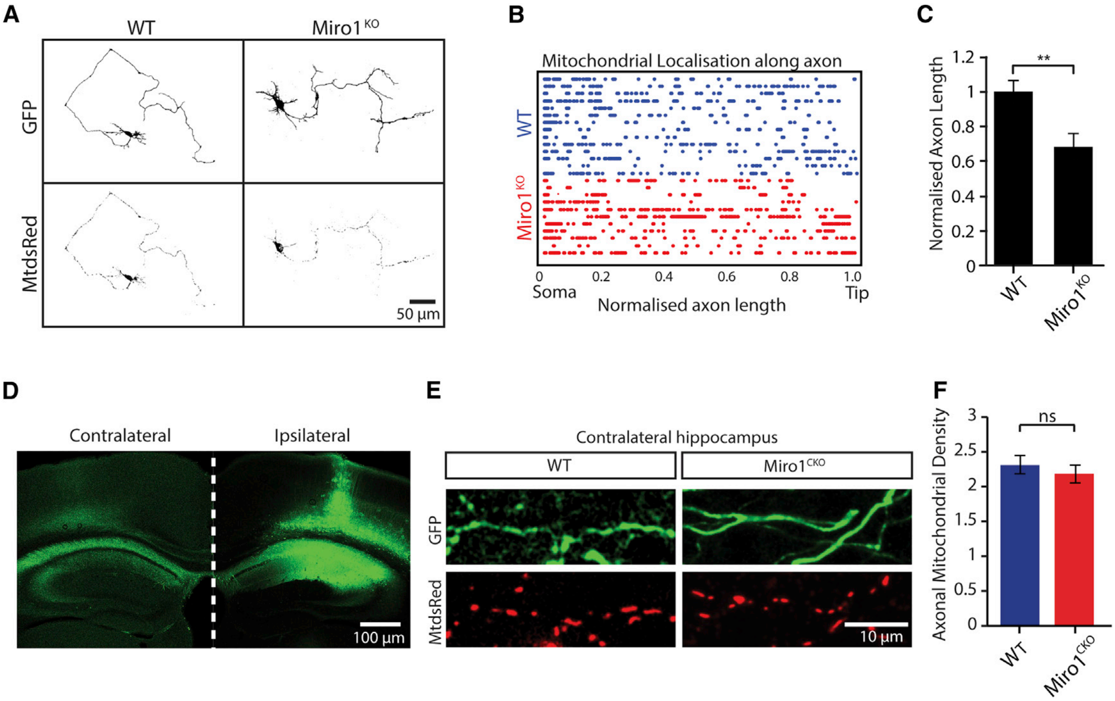

Figure 4. Mitochondrial Distribution along the Axon Is Independent of Miro1

(A) Stitched images (grayscale) showing the whole axonal length of WT and Miro1^{KO} neurons at 6 DIV (scale bar, 50 μm).

(B) Plot of the relative positions of individual mitochondria over length-normalized axons.

(C) Total axonal length from WT and Miro1^{KO} neurons (number of neurons, WT = 14, Miro1^{KO} = 11, t test).

(D) Images of the contralateral and ipsilateral hemispheres of AAV2-injected brains.

(E) Distribution of axonal mitochondria in the contralateral hippocampus from CA3 neurons infected with AAV2 encoding GFP and MtdsRed2.

(F) Density of axonal mitochondria in WT and Miro1^{CKO} neurons (number of axons, WT = 17, Miro1^{CKO} = 15; number of animals, WT = 2, Miro1^{CKO} = 2).

$p < 0.05$, **$p < 0.01$, ***$p < 0.001$. Error bars are SEM.

of neurons, detected by quantification of NeuN staining in these regions (Figures 5A, 5B, and S5F). However, the loss of dendritic complexity at this time point was accompanied by a 2-fold increase in GFAP signal ($p < 0.01$) and infiltration of reactive astrocytes, possibly as a protection mechanism to scavenge and clear cellular material product from the initial distal dendritic loss (Figures 5A, 5E, and 5F). At a later time point, 12 months, Miro1^{CKO} animals showed an enhanced loss of the dendritic tree in CA1 pyramidal neurons and a marked decrease in the number of neurons compared to WT brains (Figures 5C, 5D, 5G, and S5F).

Accordingly, the absence of Miro1-mediated mitochondrial positioning leads to a progressive dendritic loss that eventually results in neuronal degeneration.

DISCUSSION

Using constitutive and conditional mouse knockout strategies we demonstrate that Miro1 is the main regulator of mitochondrial transport and localization, important for ensuring correct dendritic development, maintenance, and neuronal survival. Miro1^{KO} neurons have less anterogradely and retrogradely

Figure 3. Requirement of Miro1 for Dendritic Maintenance

(A and B) Mitochondrial distribution in WT and Miro1^{CKO} CA1 neurons at 4 and 8 months of age (A) and normalized mitochondrial index (B) (n = images: at 4 months WT = 23, Miro1^{CKO} = 23; at 8 months WT = 22, Miro1^{CKO} = 20; t test).

(C and D) Dendritic segments from the stratum lacunosum-moleculare of AAV infected animals at 4 months (C). Quantification of the dendritic length not occupied by mitochondria (D) in WT and Miro1^{CKO}.

(E–J) Reconstructed traces (E), branchpoint Sholl analysis (F), dendritic length (G), and number of branchpoints (H) from WT and Miro1^{CKO} Golgi-stained CA1 neurons at 8 months of age. (I) Normalized BP^{90} value for branching along the apical dendrite. (J) Subtracted (WT-Miro1^{CKO}) plot of the Sholl distribution (n = cells: WT = 15, Miro1^{CKO} = 14; ANOVA-NK).

(K) FluoroNissl staining of brain coronal sections showing the hippocampus, cortex, and lateral ventricles of 8-month-old WT and Miro1^{CKO} brains (scale bar, 500 μm). Right panels show the structure of the cerebral cortex in the somatosensory area (scale bar, 100 μm).

(L and M) Quantification of hippocampal (yellow arrows) (L) and cortical thickness (M) from brains at 8 months (n = animals: WT = 4, Miro1^{CKO} = 4, Miro2^{KO} = 4; ANOVA-NK).

*$p < 0.05$, **$p < 0.01$, ***$p < 0.001$. Error bars are SEM.

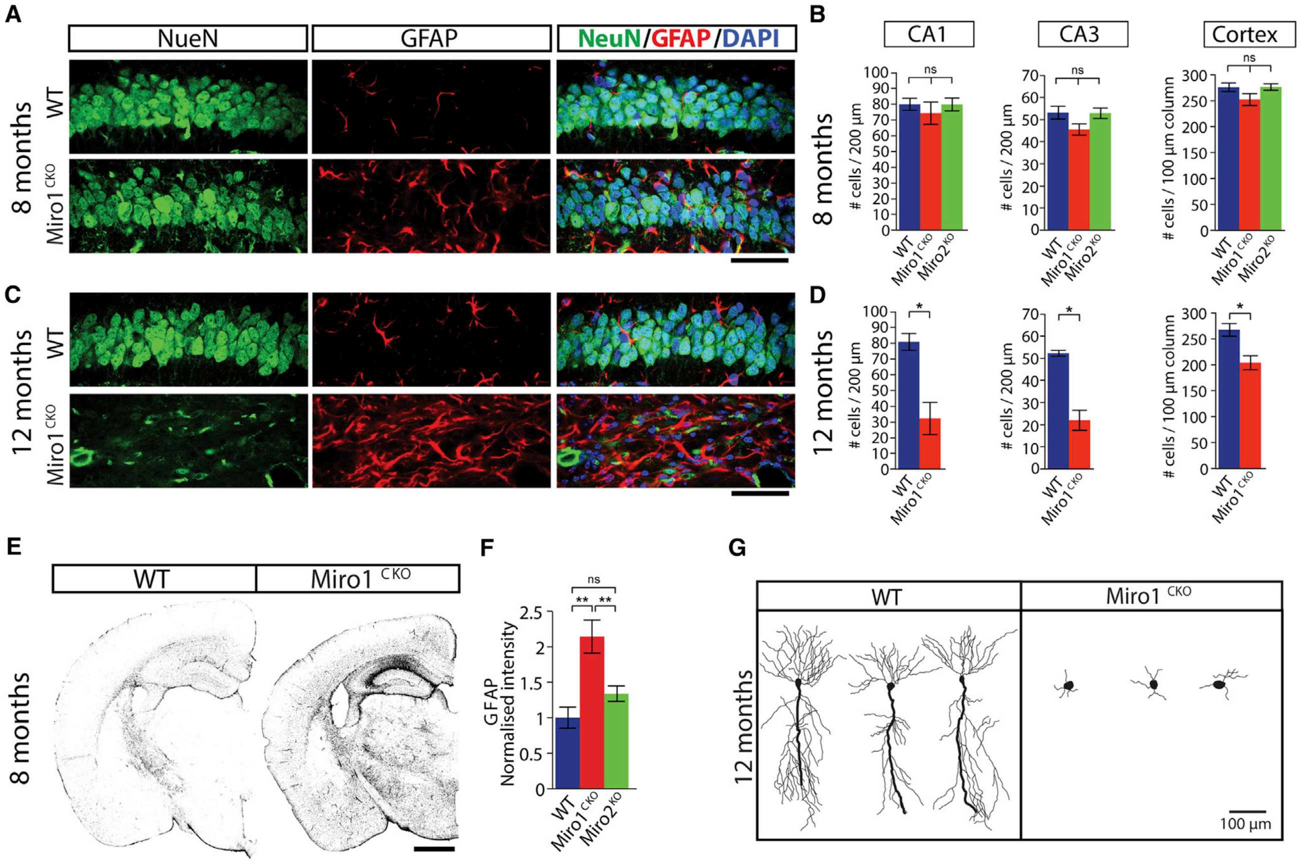

Figure 5. Miro1 Deletion in Mature Neurons Leads to Neurodegeneration

(A and C) Images of the CA1 region of the hippocampus from WT and Miro1^{CKO} animals at 8 (A) and 12 (C) months of age. Neuronal marker NeuN (green), glial marker GFAP (red), and nuclear staining DAPI (blue) (scale bar, 50 μm).

(B and D) Number of NeuN positive cells in 200-μm-width fields of CA1 and CA3 regions and 100 μm width columns from the somatosensory area of the cortex from WT, Miro1^{CKO}, and Miro2^{KO} animals at 8 (B) and 12 (D) months.

(E and F) Confocal images of hemibrains immunostained with GFAP (grayscale) of WT and Miro1^{CKO} animals at 8 months (E) (scale bar, 1 mm). Normalized GFAP intensity measured on hemibrains (F) at 8 months of age from WT, Miro1^{CKO}, and Miro2^{KO} animals (n = animals, 8 months WT = 4, Miro1^{CKO} = 4, Miro2^{KO} = 4; ANOVA-NK).

(G) Golgi-stained neurons from the CA1 region of WT and Miro1^{CKO} brains at 12 months of age.

*$p < 0.05$, **$p < 0.01$, ***$p < 0.001$. Error bars are SEM.

moving mitochondria in both dendrites and axons, whereas knocking out Miro2 has no significant effect suggesting that either Miro1 can compensate for loss of Miro2 or that Miro2 has evolved other roles for mitochondrial function. Loss of Miro1 leads to accumulation of mitochondria in proximal dendrites, resulting in neurons with shorter dendrites but with significantly increased branching within proximal regions. Several earlier studies have supported links between mitochondrial function and neuronal development (Ishihara et al., 2009; Norkett et al., 2016; van Spronsen et al., 2013). Disrupting the mitochondrial fission and fusion machinery (e.g., by targeting mitofusins or Drp1) disrupts dendritic growth (Chen et al., 2007; Ishihara et al., 2009), but it is hard to separate effects on mitochondrial dynamics, function, and trafficking in these experiments, given that targeting the fission/fusion machinery can lead to depolarized mitochondria with altered genetic content (Chen et al., 2007; MacAskill and Kittler, 2010). By directly targeting the Miro trafficking machinery, we have avoided the potential non-mitochondrial effects of targeting the TRAKs (Birsa et al., 2013; Kimura and Murakami, 2014; van Spronsen et al., 2013), the kinesin/dynein complexes (Tanaka et al., 1998), or artificially clustering mitochondria with the Golgi (Fukumitsu et al., 2015), which also has a critical role in dendritic morphogenesis (Horton et al., 2005). Interestingly, a similar effect is seen in vivo upon conditional CaMKIIα-Cre-driven, postnatal deletion of Miro1 in hippocampus and cortex demonstrating that the relationship between mitochondrial positioning and dendritic branch distribution plays a direct role in sculpting and maintaining mature neuronal circuits.

Remarkably mitochondrial distribution into axons is preserved in cultured neurons that lack Miro1 despite a notable 65% decrease of fast axonal mitochondrial trafficking. This is in stark contrast to the significant depletion of mitochondria from distal

dendrites (which have a similar microtubule polarization as axons [Baas et al., 1988]) suggesting that different mechanisms are involved in the localization of mitochondria within axons and dendrites independent on microtubule orientation. We show that Miro2 overexpression can, to a small extent, rescue axonal mitochondrial trafficking in Miro1^{KO} cultured neurons. The increase in Miro2 protein levels in Miro1^{CKO} hippocampus may compensate the lack of Miro1 in these neurons and could explain the slow progression of the neurodegeneration phenotype and/or why Miro1^{CKO} axons are still populated with mitochondria. Coupling of mitochondria to cytoskeletal transport pathways via a Miro1-independent mechanism may also still occur, for example via Miro2 or an unknown TRAK1 adaptor (van Spronsen et al., 2013) or through the mitochondrial motor Kif1b (Nangaku et al., 1994). The small amount of remaining mitochondria transported in Miro1^{KO} axons may be anchored by axonal syntaphilin (Kang et al., 2008) or by mitochondrial presynaptic capture (Courchet et al., 2013). The decreased motility of stabilized mitochondria in mutant axons may nonetheless impact mitochondrial dynamics and turnover resulting in mitochondria being less able to support axonal growth or branching (Spillane et al., 2013).

Conditional deletion of Miro1 by enolase2 promoter-driven Cre was recently proposed to cause axonal pathology in juvenile mice similar to an upper motor neuron disease (Nguyen et al., 2014). This genetic lesion is expected to affect multiple populations of neurons in brain, spinal cord, and peripheral nervous system starting as early in development as E13.5 (Kwon et al., 2006). In contrast, here we show that conditional deletion of Miro1 in mature neurons drives the loss of dendritic complexity, which is clearly evident 8 months after birth and is followed by neuronal loss (that is evident at 12 months).

A number of neurological and neurodegenerative diseases have been related to mitochondrial dysfunction and disruption of mitochondrial trafficking (Birsa et al., 2013; MacAskill and Kittler, 2010; Sheng and Cai, 2012), but the mechanisms underlying the pathophysiology of such disorders have frequently been difficult to identify. Axons are up to 10^4 times longer than dendrites and have often been the focus of attention when studying neurological diseases associated with mutations in transport components (Millecamps and Julien, 2013). Our study cannot rule out an axonal component participating in the neurodegenerative phenotype observed in Miro1^{CKO} animals. However, our Rab7GFP trafficking experiments suggesting that trophic support is not affected and our analysis of mitochondrial distribution in axons from 8-month-old Miro1^{CKO} animals suggest that an axonal pathology is unlikely the origin of the degeneration phenotype. The idea that alterations in axonal transport (mitochondrial and other organelles) are either a necessary or sufficient causative factor in the neurodegenerative progression has recently been challenged, supporting the idea that axonal transport defects and axonal degeneration may course independently of each other (Marinkovic et al., 2012). It is well known, however, that the loss of dendritic complexity is a hallmark of a number of neurological and neurodegenerative diseases (Grutzendler et al., 2007; Kulkarni and Firestein, 2012). Furthermore, recent work in a mouse model of Alzheimer's disease establishes a mechanistic link between dendritic structural degeneration and neuronal hyperexcitability resulting from changes in the electrical properties of the affected neurons and that may apply to other neurodegenerative diseases where changes in dendritic morphology occur (Šišková et al., 2014).

Altogether we demonstrate that Miro1 function is critical in regulating the development of the dendritic tree and in maintaining and sustaining arborization. Mature neurons lacking Miro1 inevitably lose their dendritic complexity and consequently their connectivity rendering them vulnerable to undergo a neurodegenerative process. With our work, we highlight the imperative need of a correct mitochondrial distribution in dendrites to sustain their architectural complexity and to protect against neurodegeneration. This pathomechanism may contribute to the neurodegenerative outcome of many human neurological diseases providing a cellular target of intervention for future therapeutic strategies.

EXPERIMENTAL PROCEDURES

Stereotaxic Hippocampal Infection

Stereotaxic viral injections were performed on 3- and 7-month-old mice as described previously (MacAskill et al., 2014). Anesthetized animals were mounted on a stereotaxic apparatus, and the brain was exposed to 7 × 40-nl measures of AAV2-CAG-MtdsRed-ires-GFP (titer = 1.2×10^{12} genome copies [GC]/mL, UPenn Vector Core [Stephen et al., 2015]) injected at coordinates relative to Bregma (Medial/Lateral, Dorsal/Ventral, Rostral/Caudal; −1.4, −1.5 to −0.5, −1.8). Expression occurred in the injected brain region for approximately 4 weeks until tissues were obtained (Figure 4D). All experimental procedures were carried out in accordance with institutional animal welfare guidelines and licensed by the UK Home Office in accordance with the Animals (Scientific Procedures) Act 1986.

Golgi Staining

Golgi staining of neurons was performed using the Rapid Golgi Stain Kit (FD NeuroTechnologies) following the manufacturer's protocol. Neurons were imaged using a 20× objective and analyzed using Neurolucida software (MBF Bioscience).

Image Acquisition and Analysis

Mitochondrial and Endosomal Trafficking

Mitochondria or Rab7GFP vesicles in neurons were live imaged with an Olympus BX60M upright microscope and 63× objective. Movies were processed in ImageJ, and transport parameters were obtained from the kymographs as previously described (Deinhardt et al., 2006; López-Doménech et al., 2012).

Sholl and Mitochondrial-Sholl Analysis

Confocal images were acquired on a Zeiss LSM700 upright confocal microscope using a 63× objective. Dendrite tracing and analysis were performed in Neuronstudio (Norkett et al., 2016). Mitochondrial-Sholl analysis was performed using a custom ImageJ plugin, which quantified the amount of MtdsRed2 pixels within shells radiating out from the soma at one-pixel intervals.

Statistical Analysis

Excel software (Microsoft) and GraphPad Prism (GraphPad) were used to analyze the data. Student's t test or Mann-Whitney test was used to test differences between two conditions. Comparison of multiple conditions with normally distributed data was performed by one-way ANOVA followed by post hoc Newman-Keuls test and for non-parametric data by Kruskal-Wallis test followed by post hoc Dunn's correction. Statistical significance was fixed at $p < 0.05$, represented as *$p < 0.05$, **$p < 0.01$, and ***$p < 0.001$. All values in text are given as average ± SEM. Error bars are SEM.

Additional details regarding animal strains, stereotaxic brain infections, neuronal culture and transfection, histology, immunoblotting, immunofluorescence, and image acquisition and analysis can be found in the Supplemental Experimental Procedures section.

SUPPLEMENTAL INFORMATION

Supplemental Information includes Supplemental Experimental Procedures and five figures and can be found with this article online at http://dx.doi.org/10.1016/j.celrep.2016.09.004.

AUTHOR CONTRIBUTIONS

G.L.-D., N.F.H., A.F.M., and J.T.K. designed the project and the experiments; G.L.-D., N.F.H., V.V., H.R., I.L.A.-C., and A.F.M. performed experiments. G.L.-D., N.F.H., and V.V. analyzed the data. G.L.-D., N.F.H., and J.T.K. wrote the manuscript.

ACKNOWLEDGMENTS

J.T.K. received funding from, ERC starting grant 282430, the Wellcome Trust, the Lister Institute, and the MRC. N.F.H. was supported by an MRC studentship, and V.V. was supported by the Wellcome Trust 4-year PhD in Neuroscience at UCL. A.F.M was supported by a Sir Henry Dale Fellowship jointly funded by the Wellcome Trust and the Royal Society (grant number 109360/Z/15/Z) and by a UCL Excellence Fellowship.

Received: July 8, 2015
Revised: June 14, 2016
Accepted: August 30, 2016
Published: October 4, 2016

REFERENCES

Baas, P.W., Deitch, J.S., Black, M.M., and Banker, G.A. (1988). Polarity orientation of microtubules in hippocampal neurons: Uniformity in the axon and nonuniformity in the dendrite. Proc. Natl. Acad. Sci. USA *85*, 8335–8339.

Birsa, N., Norkett, R., Higgs, N., Lopez-Domenech, G., and Kittler, J.T. (2013). Mitochondrial trafficking in neurons and the role of the Miro family of GTPase proteins. Biochem. Soc. Trans. *41*, 1525–1531.

Chen, H., McCaffery, J.M., and Chan, D.C. (2007). Mitochondrial fusion protects against neurodegeneration in the cerebellum. Cell *130*, 548–562.

Courchet, J., Lewis, T.L., Jr., Lee, S., Courchet, V., Liou, D.Y., Aizawa, S., and Polleux, F. (2013). Terminal axon branching is regulated by the LKB1-NUAK1 kinase pathway via presynaptic mitochondrial capture. Cell *153*, 1510–1525.

Deinhardt, K., Salinas, S., Verastegui, C., Watson, R., Worth, D., Hanrahan, S., Bucci, C., and Schiavo, G. (2006). Rab5 and Rab7 control endocytic sorting along the axonal retrograde transport pathway. Neuron *52*, 293–305.

Eyal, G., Mansvelder, H.D., de Kock, C.P., and Segev, I. (2014). Dendrites impact the encoding capabilities of the axon. J. Neurosci. *34*, 8063–8071.

Fransson, S., Ruusala, A., and Aspenström, P. (2006). The atypical Rho GTPases Miro-1 and Miro-2 have essential roles in mitochondrial trafficking. Biochem. Biophys. Res. Commun. *344*, 500–510.

Fukumitsu, K., Fujishima, K., Yoshimura, A., Wu, Y.K., Heuser, J., and Kengaku, M. (2015). Synergistic action of dendritic mitochondria and creatine kinase maintains ATP homeostasis and actin dynamics in growing neuronal dendrites. J. Neurosci. *35*, 5707–5723.

Grutzendler, J., Helmin, K., Tsai, J., and Gan, W.B. (2007). Various dendritic abnormalities are associated with fibrillar amyloid deposits in Alzheimer's disease. Ann. N Y Acad. Sci. *1097*, 30–39.

Harris, J.J., Jolivet, R., and Attwell, D. (2012). Synaptic energy use and supply. Neuron *75*, 762–777.

Horton, A.C., Rácz, B., Monson, E.E., Lin, A.L., Weinberg, R.J., and Ehlers, M.D. (2005). Polarized secretory trafficking directs cargo for asymmetric dendrite growth and morphogenesis. Neuron *48*, 757–771.

Ishihara, N., Nomura, M., Jofuku, A., Kato, H., Suzuki, S.O., Masuda, K., Otera, H., Nakanishi, Y., Nonaka, I., Goto, Y., et al. (2009). Mitochondrial fission factor Drp1 is essential for embryonic development and synapse formation in mice. Nat. Cell Biol. *11*, 958–966.

Jan, Y.N., and Jan, L.Y. (2010). Branching out: Mechanisms of dendritic arborization. Nat. Rev. Neurosci. *11*, 316–328.

Kabaso, D., Coskren, P.J., Henry, B.I., Hof, P.R., and Wearne, S.L. (2009). The electrotonic structure of pyramidal neurons contributing to prefrontal cortical circuits in macaque monkeys is significantly altered in aging. Cereb. Cortex *19*, 2248–2268.

Kang, J.S., Tian, J.H., Pan, P.Y., Zald, P., Li, C., Deng, C., and Sheng, Z.H. (2008). Docking of axonal mitochondria by syntaphilin controls their mobility and affects short-term facilitation. Cell *132*, 137–148.

Kimura, T., and Murakami, F. (2014). Evidence that dendritic mitochondria negatively regulate dendritic branching in pyramidal neurons in the neocortex. J. Neurosci. *34*, 6938–6951.

Kulkarni, V.A., and Firestein, B.L. (2012). The dendritic tree and brain disorders. Mol. Cell. Neurosci. *50*, 10–20.

Kuzawa, C.W., Chugani, H.T., Grossman, L.I., Lipovich, L., Muzik, O., Hof, P.R., Wildman, D.E., Sherwood, C.C., Leonard, W.R., and Lange, N. (2014). Metabolic costs and evolutionary implications of human brain development. Proc. Natl. Acad. Sci. USA *111*, 13010–13015.

Kwon, C.H., Zhou, J., Li, Y., Kim, K.W., Hensley, L.L., Baker, S.J., and Parada, L.F. (2006). Neuron-specific enolase-cre mouse line with cre activity in specific neuronal populations. Genesis *44*, 130–135.

López-Doménech, G., Serrat, R., Mirra, S., D'Aniello, S., Somorjai, I., Abad, A., Vitureira, N., García-Arumí, E., Alonso, M.T., Rodriguez-Prados, M., et al. (2012). The Eutherian Armcx genes regulate mitochondrial trafficking in neurons and interact with Miro and Trak2. Nat. Commun. *3*, 814.

MacAskill, A.F., and Kittler, J.T. (2010). Control of mitochondrial transport and localization in neurons. Trends Cell Biol. *20*, 102–112.

Macaskill, A.F., Rinholm, J.E., Twelvetrees, A.E., Arancibia-Carcamo, I.L., Muir, J., Fransson, A., Aspenstrom, P., Attwell, D., and Kittler, J.T. (2009). Miro1 is a calcium sensor for glutamate receptor-dependent localization of mitochondria at synapses. Neuron *61*, 541–555.

MacAskill, A.F., Atkin, T.A., and Kittler, J.T. (2010). Mitochondrial trafficking and the provision of energy and calcium buffering at excitatory synapses. Eur. J. Neurosci. *32*, 231–240.

MacAskill, A.F., Cassel, J.M., and Carter, A.G. (2014). Cocaine exposure reorganizes cell type- and input-specific connectivity in the nucleus accumbens. Nat. Neurosci. *17*, 1198–1207.

Marinkovic, P., Reuter, M.S., Brill, M.S., Godinho, L., Kerschensteiner, M., and Misgeld, T. (2012). Axonal transport deficits and degeneration can evolve independently in mouse models of amyotrophic lateral sclerosis. Proc. Natl. Acad. Sci. USA *109*, 4296–4301.

Millecamps, S., and Julien, J.P. (2013). Axonal transport deficits and neurodegenerative diseases. Nat. Rev. Neurosci. *14*, 161–176.

Nangaku, M., Sato-Yoshitake, R., Okada, Y., Noda, Y., Takemura, R., Yamazaki, H., and Hirokawa, N. (1994). KIF1B, a novel microtubule plus end-directed monomeric motor protein for transport of mitochondria. Cell *79*, 1209–1220.

Nguyen, T.T., Oh, S.S., Weaver, D., Lewandowska, A., Maxfield, D., Schuler, M.H., Smith, N.K., Macfarlane, J., Saunders, G., Palmer, C.A., et al. (2014). Loss of Miro1-directed mitochondrial movement results in a novel murine model for neuron disease. Proc. Natl. Acad. Sci. USA *111*, E3631–E3640.

Norkett, R., Modi, S., Birsa, N., Atkin, T.A., Ivankovic, D., Pathania, M., Trossbach, S.V., Korth, C., Hirst, W.D., and Kittler, J.T. (2016). DISC1-dependent regulation of mitochondrial dynamics controls the morphogenesis of complex neuronal dendrites. J. Biol. Chem. *291*, 613–629.

Rangaraju, V., Calloway, N., and Ryan, T.A. (2014). Activity-driven local ATP synthesis is required for synaptic function. Cell *156*, 825–835.

Sheng, Z.H., and Cai, Q. (2012). Mitochondrial transport in neurons: Impact on synaptic homeostasis and neurodegeneration. Nat. Rev. Neurosci. *13*, 77–93.

Šišková, Z., Justus, D., Kaneko, H., Friedrichs, D., Henneberg, N., Beutel, T., Pitsch, J., Schoch, S., Becker, A., von der Kammer, H., and Remy, S. (2014). Dendritic structural degeneration is functionally linked to cellular hyperexcitability in a mouse model of Alzheimer's disease. Neuron *84*, 1023–1033.

Skarnes, W.C., Rosen, B., West, A.P., Koutsourakis, M., Bushell, W., Iyer, V., Mujica, A.O., Thomas, M., Harrow, J., Cox, T., et al. (2011). A conditional knockout resource for the genome-wide study of mouse gene function. Nature *474*, 337–342.

Spillane, M., Ketschek, A., Merianda, T.T., Twiss, J.L., and Gallo, G. (2013). Mitochondria coordinate sites of axon branching through localized intra-axonal protein synthesis. Cell Rep. *5*, 1564–1575.

Spruston, N. (2008). Pyramidal neurons: Dendritic structure and synaptic integration. Nat. Rev. Neurosci. *9*, 206–221.

Stephen, T.L., Higgs, N.F., Sheehan, D.F., Al Awabdh, S., López-Doménech, G., Arancibia-Carcamo, I.L., and Kittler, J.T. (2015). Miro1 regulates activity-driven positioning of mitochondria within astrocytic processes apposed to synapses to regulate intracellular calcium signaling. J. Neurosci. *35*, 15996–16011.

Tanaka, Y., Kanai, Y., Okada, Y., Nonaka, S., Takeda, S., Harada, A., and Hirokawa, N. (1998). Targeted disruption of mouse conventional kinesin heavy chain, kif5B, results in abnormal perinuclear clustering of mitochondria. Cell *93*, 1147–1158.

van Spronsen, M., Mikhaylova, M., Lipka, J., Schlager, M.A., van den Heuvel, D.J., Kuijpers, M., Wulf, P.S., Keijzer, N., Demmers, J., Kapitein, L.C., et al. (2013). TRAK/Milton motor-adaptor proteins steer mitochondrial trafficking to axons and dendrites. Neuron *77*, 485–502.

Cell Reports, Volume *17*

Supplemental Information

Loss of Dendritic Complexity Precedes Neurodegeneration in a Mouse Model with Disrupted Mitochondrial Distribution in Mature Dendrites

Guillermo López-Doménech, Nathalie F. Higgs, Victoria Vaccaro, Hana Roš, I. Lorena Arancibia-Cárcamo, Andrew F. MacAskill, and Josef T. Kittler

Supplementary material

Supplementary experimental procedures

Animals

The *Rhot1* (MBTN_ EPD0066_2_F01; Allele: *Rhot1*$^{tm1a(EUCOMM)Wtsi}$) and *Rhot2* (MCSF_ EPD0389_5_A05; *Rhot2*$^{tm1(KOMP)Wtsi}$) mice lines were obtained from the Wellcome Trust Sanger Institute as part of the International Knockout Mouse Consortium (IKMC) (Skarnes et al., 2011). The *Rhot1* transgenic line was generated following the Knockout-First strategy on C57BL/6J-Tyr^{cBrd} and subsequently backcrossed in C57BL/6N Taconic Denmark strain. The *Rhot2* transgenic line was generated as a reporter-tagged deletion on C57BL/6N Taconic USA background. CAMKIIα-CRE strain has been described previously (Mantamadiotis et al., 2002). Animals were maintained under controlled conditions (temperature 20 ± 2°C; 12 hour light-dark cycle). Food and water were provided *ad libitum*. The genotyping was carried out following Sanger's recommended procedures, briefly the DNA was extracted from ear biopsies and PCRs were performed with the following primers (5' to 3'): Rhot1_16_F: TTAGGATTTGTACTTTGCCCCTG; Rhot1_16_R: AAAACCCTTCCTGCATCACC; Rhot2_WT_F3: GGCGTTAGCTCCAGTGAGTC; Rhot2_WT_R3: AGGATGTGAAGAGGGTGGTG; Rhot2_mut_F4: GGGTTGAAGGAGAGGGTTG; CAS_R1_Term: TCGTGGTATCGTTATGCGCC; LacZ_2_small_F: ATCACGACGCGCTGTATC; LacZ_2_small_R: ACATCGGGCAAATAATATCG. To test for CRE recombination we designed specific primers flanking the loxP sites: Rhot1_Ex2G_F: GGAGTAGAGAAGTCAGATTCCAG and Rhot1_Ex2G_R: GAAGGCGTCAGATCACATTG. All experimental procedures were carried out in accordance with institutional animal welfare guidelines and licensed by the UK Home Office in accordance with the Animals (Scientific Procedures) Act 1986.

Stereotaxic hippocampal infection

Viral injections were performed on mice as described previously (Cetin et al., 2006; MacAskill et al., 2014). Stereotaxic injections were performed on mice at 3 and 7 months respectively, which were anaesthetized with ketamine (100 mg / kg) and xylazine (5 mg / kg) administered intraperitoneally (i.p.). Animals were gently mounted on a stereotaxic apparatus and a single incision was made along the midline to reveal the skull. The head was leveled and injection coordinates determined relative to bregma (Medial / Lateral, Dorsal / Ventral, Rostral / Caudal; BLA: -1.4, -1.5 to -0.5, -1.8). Long-shaft borosilicate pipettes with a tip diameter of 5 to 10 µm were backfilled with 0.8 µl of AAV2-CAG-MtdsRed-ires-GFP (titer = 1.2 x 10^{12} GC / ml, UPenn Vector Core, (Stephen et al., 2015)). A small hole was created in the skull over the injection site using a fine dental drill and 7 x 40 nl measures of virus were injected at 3 different points over a 1 mm range resulting in a final injection volume of no more than 300 nl. The pipette was left in place for an additional 5 min to minimize diffusion and then slowly removed. The wound was sutured and sealed and the mice monitored until recovery on a heat pad before returning to their home cage. Expression occurred in the injected brain region for approximately four weeks until tissues were obtained (Figure 4D).

Cell culture and transfection

Hippocampal cultures were obtained from E16 mouse brains from crosses of heterozygous Miro1 (or Miro2) animals. After dissecting each embryo a sample from the tail was used for genotyping and the hippocampi were further processed, separated and identified at all times. Cells were seed at a density of 30,000-50,000 cells/cm^2 onto Poly-L-Lysine (0.5 mg/ml in 0.1M borate buffer, pH 8) coated coverslips and transfected at 12 DIV by calcium phosphate or Lipofectamine2000 (Invitrogen). 30% of the medium was replaced with fresh medium every 3-4 days starting at 7DIV.

DNAs, reagents and antibodies.

GFP, MtdsRed2 and Miro1-myc have been previously described (Macaskill et al., 2009). Su9-EGFP was a gift from David Chan (Addgene plasmid # 23214) and pRK5-myc-Miro2 was a gift from Pontus Aspenström (Addgene plasmid # 47891), both obtained from addgene. FluoroNissl (NeuroTrace 520/525) was purchase from Mollecular Probes (Invitrogen) and used following recommended guidelines. For immunocytochemistry and immunohistochemistry antibodies were used as follows: rat anti-GFP (1:1000, Nacalai-Tesque, GF090R); mouse anti-AnkG (1:200, NeuroMab); rabbit anti-Miro1 (1:250, Atlas Antibodies); rabbit anti-Tom20 (1:300, SantaCruz); rabbit anti-GFAP (1:1000, Dako); mouse anti-NeuN (1:300, chemicon). For westernblotting antibodies were used as follows: rabbit anti-Miro1 (1:1000, Atlas Antibodies); mouse anti-Miro2 (1:1000, NeuroMab); rabbit anti-actin (1:10000, Sigma), mouse ApoTrak-cocktail (1:1000, MitoSciences). Horse radish peroxidase-conjugated anti-mouse or anti-rabbit IgGs where used at 1:10000 (Rockland).

Western blotting and immunocytochemistry

Hippocampal cultures where fixed when required in 4% PFA for 10 min at RT and rinsed several times in PBS. For immunocytochemistry coverslips where permeabilized in PBS with 0.1% Triton-X100 and incubated 1 hour in blocking solution (1% BSA, 10% fetal bovine serum, 0.2M glycine in PBS with 0.1%Triton-X100). Primary antibodies where applied in blocking solution at the desired concentration and incubated for 2 hours. AlexaFluor (Invitrogen) secondary antibodies where incubated for one hour at 1:800 in blocking solution. Coverslips were mounted in Mowiol mounting media and kept in the dark at 4°C until imaged. For western blotting 20 μg of protein from lysates of whole E18 forebrain, or adult cortex, hippocampus or cerebellum were loaded on 8-12% acrylamide gels and transferred to nitrocellulose membranes (GE Healthcare Bio-Sciences) using the Biorad system. Membranes where blocked in 3% BSA on TBS-T or 4% powder milk in PBS-T for 1-2 hours.

Antibodies were incubated in blocking solution (overnight at 4°C for primary antibodies or 1 hour at RT for HRP-conjugated secondary antibodies). Membranes were developed using the ECL-Plus reagent (GE Healthcare Bio-Sciences) and acquired in a chemiluminescence imager coupled to a CCD camera (ImageQuant LAS 4000mini). Densitometric analysis was performed using Quantity-One software (Biorad).

Tissue processing

Animals of the selected age were culled by cervical dislocation or CO_2 exposure. Hemibrains were either snap-frozen in liquid nitrogen for protein studies, fixed in chilled 4% PFA for further processing for histology studies or immersed in Golgi solution for dendritic morphometric analysis. Animals infected with AAV encoding GFP and MtdsRed2 were anesthetized with isoflurane and transcardially perfused with chilled 4% PFA to maintain mitochondrial integrity and further processed for histology analysis.

Histology

Hemibrains were fixed by immersion in 4% PFA overnight at 4°C, cryoprotected in 30% sucrose-PBS for 24-48 hours and stored at -80°C. Tissue was serially cryosectioned in a Bright OTF-AS Cryostat (Bright Instrument, Co. Ltd.) at 30 μm thickness and stored in cryoprotective solution (30% PEG, 30% glycerol in PBS) at -20°C until used. FluoroNissl (NeuroTrace 520/525) staining was performed following manufacturer instructions. Immunohistochemistry studies were performed in free-floating sections. Briefly, tissue was washed 3-5 times in PBS over 30 minutes and permeabilized in PBS-0.5% Triton-X100 over a total of 3-5 washes during 30 minutes. Sections were then blocked at RT in a solution containing 3% BSA, 10% fetal bovine serum and 0.2M glycine in PBS 0.5% Triton-X100 for 3-4 hours. Eventually, tissue was further blocked overnight at 4°C in the same blocking solution plus purified goat anti-mouse Fab-

fragment (Jackson Immunoresearch) at a concentration of 50 μg/ml to reduce endogenous background. Sections were then further washed and incubated overnight at 4°C in primary antibodies prepared in blocking solution. After washing in PBS 0.5% Triton-X100 secondary antibodies were applied in blocking solution and incubated at RT for 3-4 hours. Sections were mounted on glass slides using Mowiol mounting media and stored at 4°C in the dark until documented.

Golgi Staining

Golgi staining of neurons was performed using the Rapid Golgi Stain Kit (FD NeuroTechnologies) following the manufactures protocol. Neurons were imaged and analyzed using Neurolucida software (MBF Bioscience) attached to an upright light widefield microscope (Pathania et al., 2014). E18 brains were left in the impregnating solution for a maximum of 5 days only and adult brains were left for 2 weeks. Following impregnation brains were sliced at 100 μm thickness using a vibratome (Leica). Within E18 brains the processes of cortical neurons were imaged using a 60x objective (Olympus, NA 1.35) and at least 10 neurons traced per a brain. The dendritic morphology of CA1 hippocampal neurons of adult brains were imaged using a 20x (Olympus, NA 0.45) objective. The entire dendritic tree of at least three neurons was traced and averaged to produce one value per animal. All animals were age matched in each time-point. $Miro1^{lox/lox,Cre(-/-)}$ (WT controls) and $Miro1^{CKO}$ animals were obtained from the same litters from each individual experiment.

Image acquisition and analysis

Mitochondrial and endosomal trafficking. Neurons were live imaged with an Olympus BX60M upright microscope using a 63X water immersion objective (NA: 1.0) and a homemade setup to allow a constant flow of 36.5°C warmed ACSF (artificial cerebrospinal fluid; 10mM HEPES, 125mM NaCl, 10mM D-Glucose, 5mM KCl, 2mM

$CaCl_2$ and 1mM $MgCl_2$ at pH7.4). For synaptic stimulation experiments 30 μM glutamate and 1 μM glycine were included in the buffer and perfused in the cultures for one minute. Images were acquired using an EM-CCD camera (Ixon; Andor, Oxford Instruments). All electronics were controlled by AndorIQ Software (Oxford Instruments) or μManager (http://micro-manager.org/wiki/Micro-Manager). Proximal axonal segments situated at least 50 μm away from the soma and principal dendrites other than the primary dendrite were selected from the GFP channel and mitochondria were imaged on the MtdsRed2 channel. Images of selected dendrites or axons were acquired every 2 seconds for a total time of 2 minutes (61 frames in total). For Rab7GFP trafficking studies movies were generated at a rate of 1 image every second for a total of 2 minutes. Generated movies were processed with ImageJ software (http://imagej.nih.gov/ij/). Alignment (stackreg) and background subtraction were applied when required. Kymographs were generated from the selection traces obtained from the GFP image. Transport parameters were obtained from the kymographs as previously described (De Vos and Sheetz, 2007; Deinhardt et al., 2006; Lopez-Domenech et al., 2012). In stimulation experiments analysis was performed in the two minutes previous to the stimulation and in the two minutes that followed the end of the stimulation. In Miro1 experiments at DIV 14 all data was obtained from nine different preparations (five of which included Miro1-myc rescue experiments). Miro2 overexpression studies in $Miro1^{KO}$ neurons were conducted over five additional independent preparations. For the $Miro2^{KO}$ experiments four different preparations were used. All 6 DIV in vitro data was obtained from 5 different experiments.

Confocal imaging. Confocal images were acquired on a Zeiss LSM700 upright confocal microscope (Carl Zeiss, Welwyn Garden City, UK) using a 63X oil immersion objective (NA: 1.4). Images were stitched together using Vias (CNIC software tools) to allow the whole dendritic arbor of the neuron to be visualised. Dendrites were then

traced using Neuronstudio (Wearne et al., 2005) and the total dendritic length, number of branch points and Sholl analysis were automatically calculated. Sholl analysis to reveal the mitochondrial distribution was performed using an ImageJ plugin designed within the lab which quantified the amount of MtdsRed2 pixels within shells radiating out from the soma at 1 pixel intervals. For histology studies confocal images were taken using a 5X (NA: 0.16) or 10X (NA: 0.3) air objective or a 63X oil immersion objective (NA: 1.4). Acquisition parameters were kept constant over experimental conditions. All histology experiments were performed with age matched animals. Three different measures from consecutive brain slices (spanning 150 to 200 μm) were taken for each independent parameter (cortical thickness, number of neurons in each region measured or GFAP intensity in hemibrains) and averaged to produce one value per animal. For mitochondrial and endosomal trafficking assays MtdsRed2 and Rab5-GFP were transfected in hippocampal neurons and simultaneously imaged at 14 DIV using a 63X water immersion objective (NA: 1.0) and a constant flow of 36.5°C warmed ACSF. Frames were obtained every 2 seconds for a total of 2 minutes. For analysis of mitochondrial distribution in AAV infected brains segments of axons (in contralateral hippocampus) or dendrites (in ipsilateral hippocampus of the infection area) of at least 50 μm length were imaged with a 63X oil immersion objective. Two animals were used per age selected and genetic condition.

Mitochondrial membrane potential in hippocampal neurons. Dissociated cortical neurons were pooled together from all E16 embryos and seeded at a density of 40.000 cells/cm^2 to serve as a supporting culture. Hippocampal cells from individual embryos were then split into 2 different tubes and nucleofected (AMAXA Biotechnology) with either GFP or CFP as reporters. Combinations of differentially nucleofected (GFP and CFP) hippocampal neurons from 2 different embryos were then seeded at a density of 5.000 cells/cm^2 (each embryo) over the supporting culture and left 14 days to develop. After genotyping, the coverslips with cocultured neurons from a WT and a KO embryo

together were selected for the experiments. These coverslips were incubated for 30 minutes at 37°C with the mitochondrial membrane potential sensor tetramethylrhodamine methyl ester (TMRM, Biotium) at 20 nM and then live imaged on a Zeiss LSM700 upright confocal microscope (Carl Zeiss, Welwyn Garden City, UK) using a 20X water immersion objective (NA: 1.0) on a constant flow of 36.5°C warmed ACSF. Stack images were taken from fields that enclosed at least one GFP transfected neuron and one CFP transfected neuron. Fluorescent intensity was measured in the somas of the transfected cells from the maximum projection images (ImageJ, http://imagej.nih.gov/ij/) and then normalized with the average intensity of at least 3 non-trasfected neurons from the supporting culture. Normalized values for each condition were obtained from 3 different experiments.

Supplementary Figure legends

Supplementary Figure S1. Related to Figure 1:

(A) *Rhot1* transgenic allele ($Rhot1^{tm1a(EUCOMM)Wtsi}$) is a knockout-first (tm1a) with the insertion of an FRT flanked L1L2_gt1 cassette between exon 1 and 2 and LoxP sites flanking exon 2. Flp driven recombination of tm1a produces *Rhot1* conditional allele (tm1c). Miro1 expression is again possible, as En2 splice acceptor has been removed. This allele has the potential to become knockout when Cre recombination occurs producing the deletion allele (tm1d).

(B) *Rhot2* allele ($Rhot2^{tm1(KOMP)Wtsi}$) is of the type tagged deletion (tm1). Exons from 2 to 16 are substituted through homology recombination by an L1L2_Bact_PCassette cassette.

(C and D) PCR analysis on DNA extracted from E16 $WT^{(+/+)}$, $Het^{(+/-)}$ and $KO^{(-/-)}$ embryos shows successful recognition of either WT and *Rhot1* mutant allele (C) or *Rhot2* deletion allele (D).

(E) Western blot analysis of Miro1 and Miro2 protein levels from E18 brain lysates from litters generated by heterozygous mattings for the *Rhot1* allele (first 6 lanes) or *Rhot2* mattings (last 5 lanes). Antibody against Miro (Atlas) recognizes both Miro1 (duplex at ~71-73 KDa) and Miro2 proteins (~74 KDa).

(F-I) Mitochondrial stopping induced by glutamate stimulation in 15 DIV $Miro1^{CKO}$ neurons and in WT are undistinguishable.

(F) Kymographs of mitochondrial movement within $Miro2^{KO}$ neurons, dendrites (left) and axons (right).

(G) Merged kymographs showing simultaneous mitochondrial movement (red) and Rab5 positive endosomal movement (green) in WT and $Miro1^{KO}$ axons.

(L-M) Rab7 positive endosome dynamics within $Miro1^{KO}$ and WT. (L) Representative kymographs showing the dynamics of Rab7 positive endosomes within the axon. (M) Quantification of the percentage of moving vesicles (WT 46.6 ± 2.4, $Miro1^{CKO}$ 45.9 ±

1.1; p=0.78, t-test) and (N) their velocities shows no difference in the dynamics of Rab7 endosomes within WT and Miro1^{KO} axons.

Supplementary Figure S2. Related to Figure 2:

(A-B).Analysis of mitochondrial transport in axons (A) and dendrites (B) in cultures at 14-15 DIV overexpressing Su9GFP or Miro2GFP. Transport analysis within the axon (A) indicates that Miro2 overexpression can only partially rescue the defects in mitochondrial transport due to the deletion of Miro1 (Data obtained from 4 independent experiments; ANOVA-NK)

(C) Mitochondrial probability map and (D) $Mito^{60}$ value (WT n=16, $Miro2^{KO}$ n=13, t-test, p=0.15), show no difference in the distribution of mitochondria within WT and $Miro2^{KO}$ dendrites.

(E-H) Quantification of dendritic morphology at 6 DIV. (E) Dendritic length, (F) number of branch points, (G) BP^{90} Branch points and (H) sholl analysis of the number of branch points shows that there is no difference in dendritic complexity between WT and $Miro2^{KO}$ neurons. (WT n=17, $Miro2^{KO}$ n=12, t-test show no significance).

Supplementary Figure S3. Related to Figure 1 and Figure 2:

(A-F) Mitochondrial trafficking analysis of 6-7 DIV neurons in axons (A-C) and dendrites (D-E). As seen in 14-15 DIV Miro1^{KO} neurons mitochondrial transport is disrupted

(G-H) At 6-7 DIV mitochondrial distribution is disrupted. (G) MPM curve and (H) the length normalised Mito60 value shows that mitochondria are accumulated towards proximal regions of neurons (WT= 12, Miro1^{KO}= 13, t-test p= 0.035). (I-L) No difference in the size of the dendritic arbors is seen in Miro1^{KO} neurons at 6-7 DIV (dendritic length: WT 796 ± 59 µm, Miro1^{KO} 760 ± 75 µm, p= 0.71; number of primary dendrites: 5.0 ± 0.40, Miro1^{KO} 5.5 ± 0.37, p= 0.40; number of branch points: WT 13 ± 1.33, Miro1^{KO} 13.8 ± 2.24, p= 0.73), however there are less branch points from 120 µm outwards from the soma, showing there is a decrease in dendritic complexity at this time-point (WT= 17, Miro1^{KO}= 18, t-test; p> 0.05, *p< 0.05)

(M) Representative image of a hippocampal neuron co-culture showing a WT neuron transfected with CFP (blue) and a Miro1^{KO} neuron transfected GFP (green) labelled with TMRM (red).

(N) Quantification of mitochondrial membrane potential. Fluorescent signal from WT and Miro1^{KO} neurons was normalized with an average measure of at least 3 neurons taken from the same image (“Ref” in graph). 30 images with one WT neuron and one Miro1^{KO} neuron were used for the analysis. One value per condition was calculated in each experiment (n= 3). One-way ANOVA followed by Newman-Keuls *post hoc* correction for multiple comparisons was applied to test significant differences between groups.

Supplementary Figure S4. Related to Figure 3 and Figure 5:

(A) Western blot analysis from 4 month adult brains, probed for Miro1, Miro2 and actin protein. Dissected regions include cortex (Ctx), hippocampus (Hipp) and cerebellum (Cereb). As in E18 embryos the antibody against Miro1 (Atlas) recognizes both Miro1 (duplex at ~71-73 KDa) and Miro2 proteins (~74 KDa).

(B) Western blot of hippocampus lysates at different stages shows that Miro1 and 2 reactivity decreases during ageing during normal conditions. At 1 month of age Miro1 deletion is already significant and Miro2 appears to be upregulated.

(C) Difference in the size of brains dissected from WT vs Miro1^{CKO} mice at 8 months.

(D-E) quantitative analysis of Miro2 levels in WT and Miro1CKO brains showing that Miro2 levels increase after Miro1 deletion.

Supplementary Figure S5. Related to Figure 3 and Figure 5:

(A) Analysis of the cortex width of Miro1^{CKO} brains compared to WT and Miro2^{KO} at 4 months (WT n= 3, Miro1^{KO} n= 3, Miro2^{KO} n= 3, Miro1/2^{DKO} n= 4)

(B-E) Morphological analysis of 4 month old WT and Miro1^{CKO} dendrites of pyramidal CA1 neurons showing that there is no difference in their dendritic morphology. (B) Reconstructed traces of CA1 neurons from 4 month old animals. Morphological quantification of the dendritic length (C) and the number of branch points (D) shows that at 4 months the dendritic architecture of Miro1^{CKO} animals is indistinguishable from WT controls (n=animals, 4 months WT n= 3, Miro1^{CKO} n= 3 and Miro2^{KO} n= 3; ANOVA and *post hoc* Newman-Keuls). (E) Sholl analysis of the number of branch points shows that there is no difference in dendritic complexity.

(F) Number of NeuN positive cells counted in cortex and CA1 and CA3 regions of the hippocampus from the corresponding genotypes at 8 and 12 months of age.

*p<0.05, **p<0.01, ***p<0.001.

Supplementary References

Cetin, A., Komai, S., Eliava, M., Seeburg, P.H., and Osten, P. (2006). Stereotaxic gene delivery in the rodent brain. Nature protocols *1*, 3166-3173.

De Vos, K.J., and Sheetz, M.P. (2007). Visualization and quantification of mitochondrial dynamics in living animal cells. Methods Cell Biol *80*, 627-682.

Deinhardt, K., Salinas, S., Verastegui, C., Watson, R., Worth, D., Hanrahan, S., Bucci, C., and Schiavo, G. (2006). Rab5 and Rab7 control endocytic sorting along the axonal retrograde transport pathway. Neuron *52*, 293-305.

Lopez-Domenech, G., Serrat, R., Mirra, S., D'Aniello, S., Somorjai, I., Abad, A., Vitureira, N., Garcia-Arumi, E., Alonso, M.T., Rodriguez-Prados, M., *et al.* (2012). The Eutherian Armcx genes regulate mitochondrial trafficking in neurons and interact with Miro and Trak2. Nature communications *3*, 814.

MacAskill, A.F., Cassel, J.M., and Carter, A.G. (2014). Cocaine exposure reorganizes cell type- and input-specific connectivity in the nucleus accumbens. Nat Neurosci *17*, 1198-1207.

Macaskill, A.F., Rinholm, J.E., Twelvetrees, A.E., Arancibia-Carcamo, I.L., Muir, J., Fransson, A., Aspenstrom, P., Attwell, D., and Kittler, J.T. (2009). Miro1 is a calcium sensor for glutamate receptor-dependent localization of mitochondria at synapses. Neuron *61*, 541-555.

Mantamadiotis, T., Lemberger, T., Bleckmann, S.C., Kern, H., Kretz, O., Martin Villalba, A., Tronche, F., Kellendonk, C., Gau, D., Kapfhammer, J., *et al.* (2002). Disruption of CREB function in brain leads to neurodegeneration. Nat Genet *31*, 47-54.

Pathania, M., Davenport, E.C., Muir, J., Sheehan, D.F., Lopez-Domenech, G., and Kittler, J.T. (2014). The autism and schizophrenia associated gene CYFIP1 is critical for the maintenance of dendritic complexity and the stabilization of mature spines. Translational psychiatry *4*, e374.

Skarnes, W.C., Rosen, B., West, A.P., Koutsourakis, M., Bushell, W., Iyer, V., Mujica, A.O., Thomas, M., Harrow, J., Cox, T., *et al.* (2011). A conditional knockout resource for the genome-wide study of mouse gene function. Nature *474*, 337-342.

Stephen, T.L., Higgs, N.F., Sheehan, D.F., Al Awabdh, S., Lopez-Domenech, G., Arancibia-Carcamo, I.L., and Kittler, J.T. (2015). Miro1 Regulates Activity-Driven Positioning of Mitochondria within Astrocytic Processes Apposed to Synapses to Regulate Intracellular Calcium Signaling. The Journal of neuroscience : the official journal of the Society for Neuroscience *35*, 15996-16011.

Wearne, S.L., Rodriguez, A., Ehlenberger, D.B., Rocher, A.B., Henderson, S.C., and Hof, P.R. (2005). New techniques for imaging, digitization and analysis of three-dimensional neural morphology on multiple scales. Neuroscience *136*, 661-680.

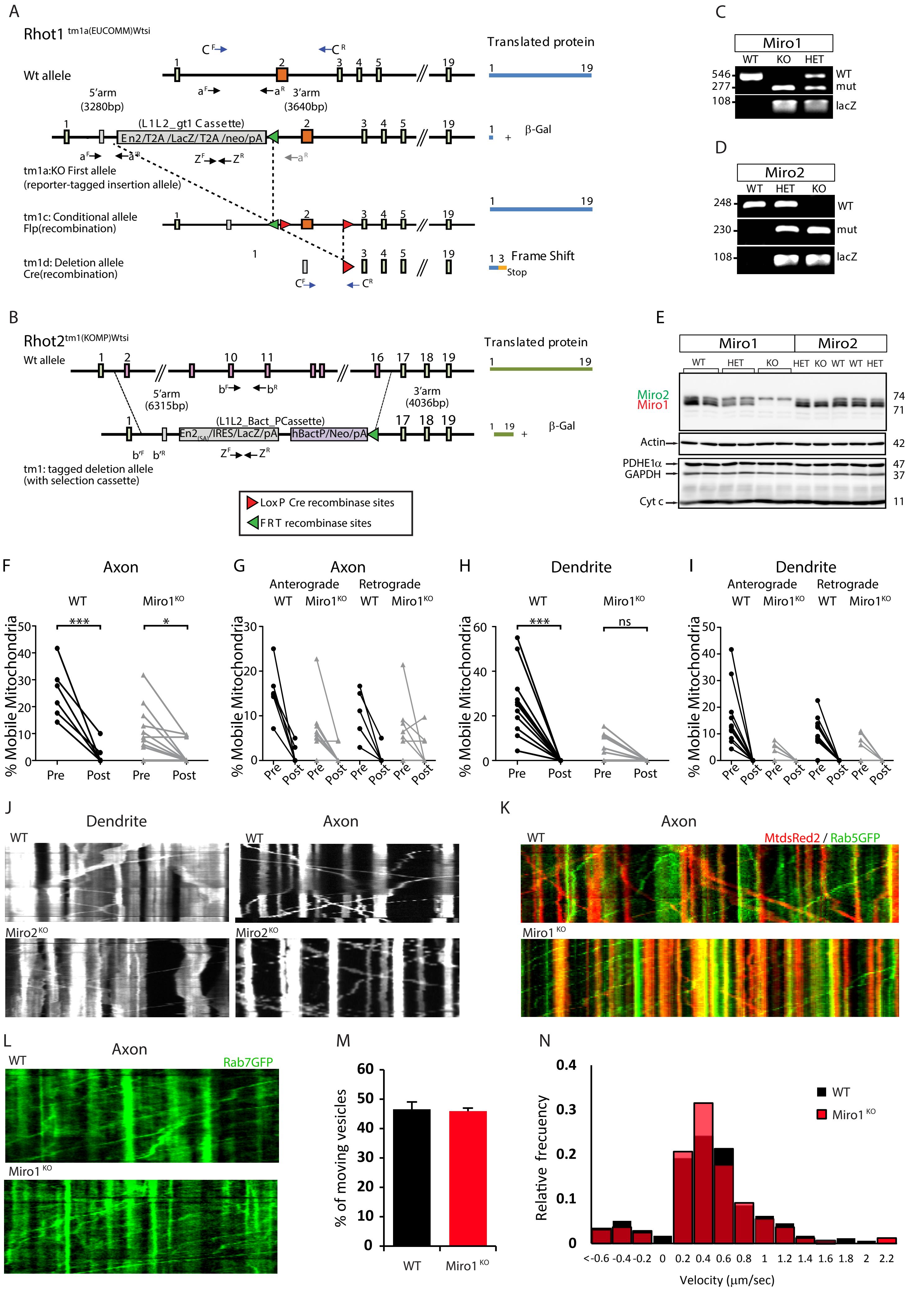

Supplementary Figure S1. Related to Figure 1

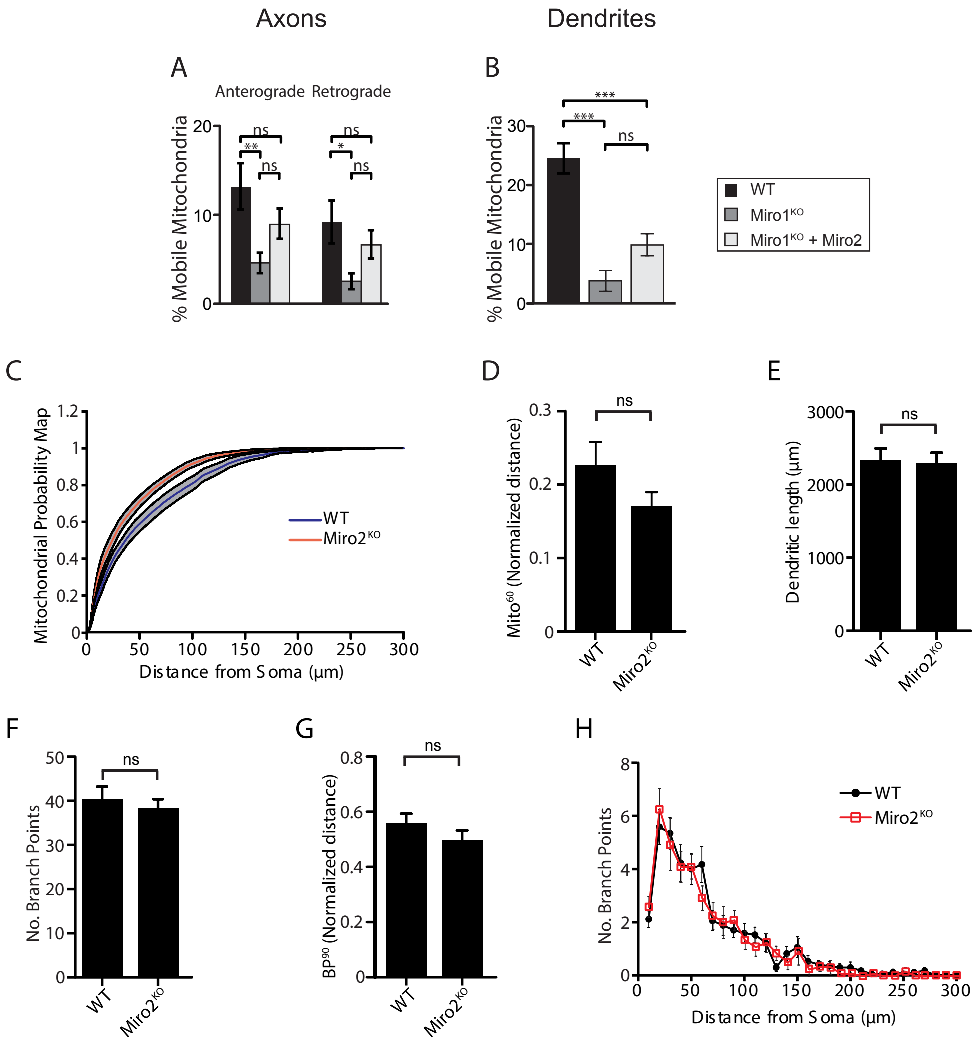

Supplementary Figure S2. Related to Figure 2

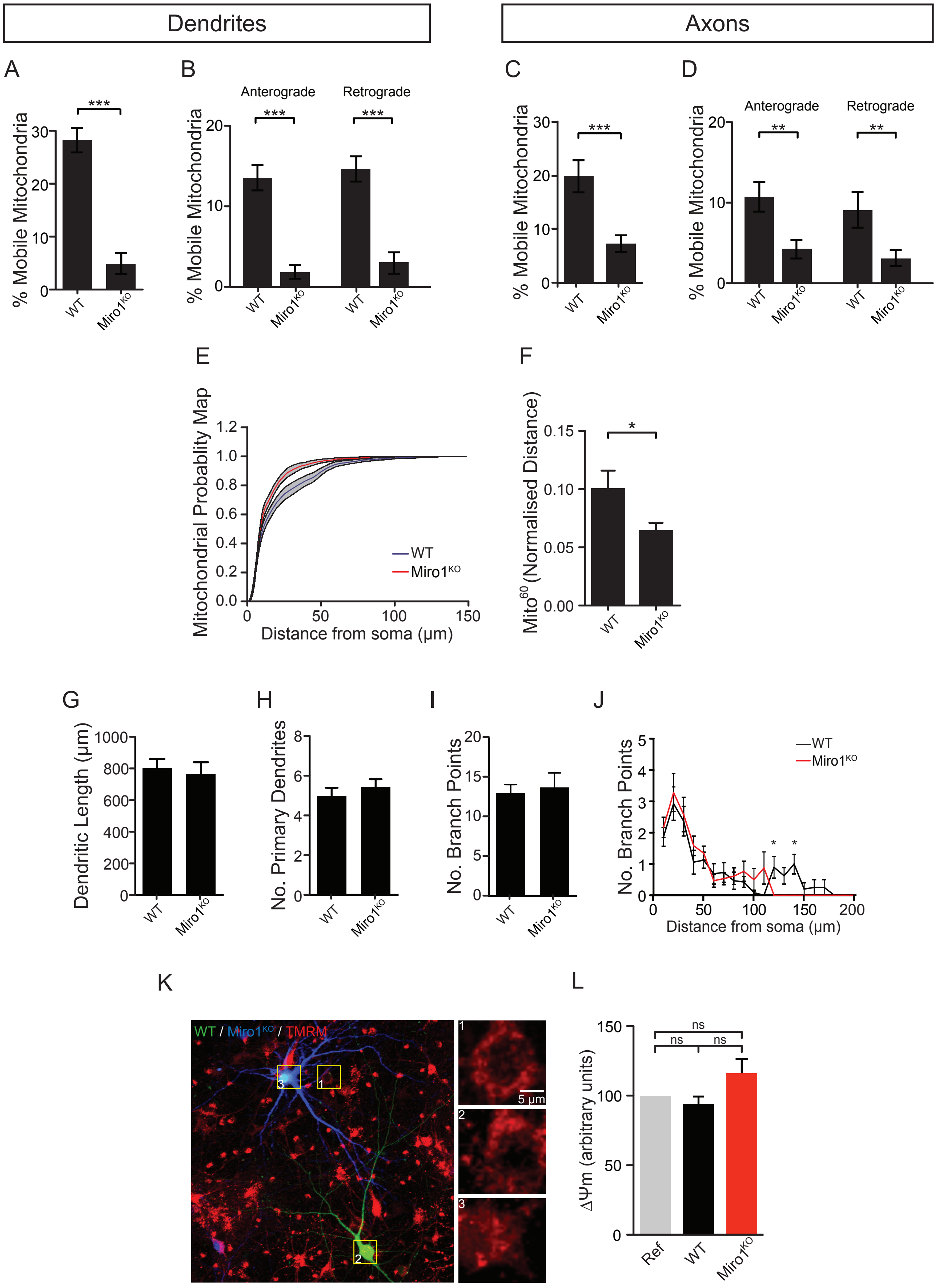

Supplementary Figure S3. Related to Figure 1 and Figure 2

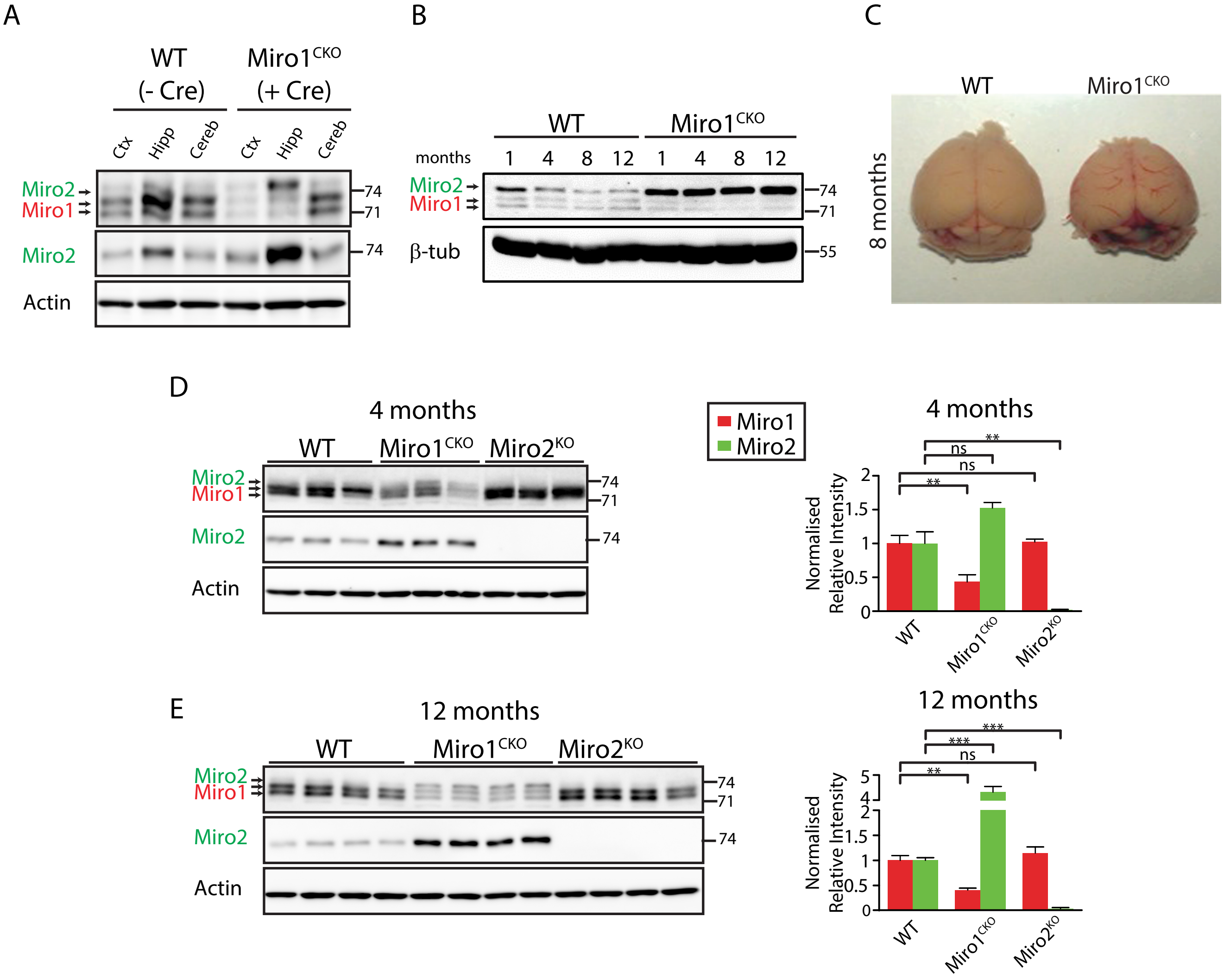

Supplementary Figure S4. Related to Figure 3 and Figure 5

A

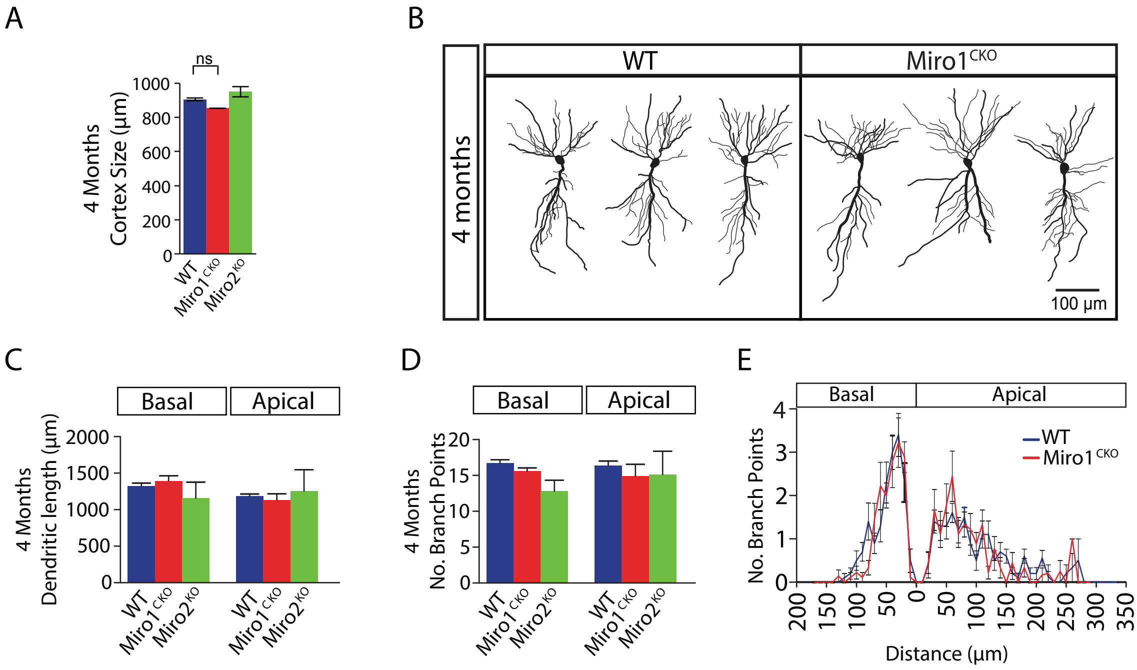

F

Genotype	Cortex (cells in 100 mm columns)	CA1 (cells in 200 mm field)	CA3 (cells in 200 mm field)
**8 months**			
Wild type	273.9 ± 7.9	79.8 ± 3.9	52.8 ± 2.8
Miro1^{CKO}	250.3 ± 11.2	74.2 ± 6.9	45.3 ± 2.5
Miro2^{KO}	274.4 ± 6.4	79.7 ± 4.2	52.6 ± 2.4
**12 months**			
Wild type	266.8 ± 12.5	80.7 ± 5.3	52.4 ± 1.3
Miro1^{CKO}	203.7 ± 13.7 (*)	32.3 ± 10.1 (*)	22.1 ± 4.6 (*)

Supplementary Figure S5. Related to Figure 3 and Figure 5
